# Immune Reactions of Vector Insects to Parasites and Pathogens

**DOI:** 10.3390/microorganisms12030568

**Published:** 2024-03-12

**Authors:** Norman Arthur Ratcliffe, Cicero Brasileiro Mello, Helena Carla Castro, Paul Dyson, Marcela Figueiredo

**Affiliations:** 1 Department of Biosciences, Swansea University, Singleton Park, Swansea SA28PP, UK; 2 Biology Institute, Universidade Federal Fluminense, Niterói 24210-130, RJ, Brazil; cicerobrasileiro@id.uff.br (C.B.M.); hcastro@id.uff.br (H.C.C.); 3 Institute of Life Science, Medical School, Swansea University, Singleton Park, Swansea SA28PP, UK; p.j.dyson@swansea.ac.uk (P.D.); marcela.figueiredo@gmail.com (M.F.)

**Keywords:** insect vector immunity, *Anopheles*, *Aedes*, *Culex*, blackflies, sandflies, tsetse flies, lice, fleas, *Rhodnius*, mosquitoes, malaria, sleeping sickness, leishmaniasis, Chagas disease, filariasis, onchocerciasis, arboviruses, eicosanoids, immune priming, phagocytosis, encapsulation, melanisation, antimicrobial peptides, pathogen recognition, signaling pathways

## Abstract

This overview initially describes insect immune reactions and then brings together present knowledge of the interactions of vector insects with their invading parasites and pathogens. It is a way of introducing this Special Issue with subsequent papers presenting the latest details of these interactions in each particular group of vectors. Hopefully, this paper will fill a void in the literature since brief descriptions of vector immunity have now been brought together in one publication and could form a starting point for those interested and new to this important area. Descriptions are given on the immune reactions of mosquitoes, blackflies, sandflies, tsetse flies, lice, fleas and triatomine bugs. Cellular and humoral defences are described separately but emphasis is made on the co-operation of these processes in the completed immune response. The paper also emphasises the need for great care in extracting haemocytes for subsequent study as appreciation of their fragile nature is often overlooked with the non-sterile media, smearing techniques and excessive centrifugation sometimes used. The potential vital role of eicosanoids in the instigation of many of the immune reactions described is also discussed. Finally, the priming of the immune system, mainly in mosquitoes, is considered and one possible mechanism is presented.

## 1. Introduction—The Good, the Bad and the Incredible about the Immune Reactions of Vector Insects against Parasites and Pathogens

The “good” thing about insect immunity is that rapid progress has been made in understanding its functional roles at the molecular level. This increased knowledge has also been applied to understanding the interaction of vector species with invading pathogens and parasites.

There are a large number of reviews in the last 10 years related to insect vector immunity including e.g., [1,2,3,4,5,6,7,8,9,10,11,12,13,14,15,16,17,18,19,20,21,22,23,24,25,26,27,28,29,30,31]. Most of these deal with a single group, genus or species of vector insects or even just with one key aspect of immunity such as the antimicrobial peptides (AMPs), signal molecules or infections caused by one particular parasite or pathogen. Herein, by way of introduction, we briefly describe the immune reactions of a range of vector insects to invading parasites and pathogens in order to gain some idea about possible common reactions and responses as a result of infection. Subsequently, many of these interactions are described with greater details in other papers in this Special Issue and should help identify where additional research is required.

The general view of vector insects is that they are all “bad” due to the diseases they transmit. There are, however, some “good” aspects of their existence. For example, although there are about 3700 species of mosquitoes [32], the majority do not transmit disease but, especially in wetlands, form key elements in the food chain of aquatic and terrestrial predators including fish, newts, bats and birds [33]. In addition, during their quest for nectar as food sources, mosquitoes are effective plant pollinators [34]. Furthermore, the saliva of blood-feeding vector insects, such as anopheline mosquitoes, tsetse flies and triatomines, produces peptidic anticoagulant molecules with unique trivalent properties to inhibit thrombin and potentially act as sources of unique drugs for treating clotting diseases in humans [35].

Despite these positive aspects of insect vectors, and as their name indicates, they are regarded as bad due to disease transmission to humans and animals resulting from adaptation of invading organisms to manipulate and overcome the vector immune defences. Actual numbers of insect vectors species are probably confined to hundreds [36], in contrast to the recently estimated 5.5 million insect species on Earth [37].

## 2. Insect Vectors of Disease

The orders Diptera and Hemiptera contain many species transmitting parasites and pathogens causing diseases such as malaria, leishmaniasis, Chagas disease, sleeping sickness, filariasis, onchocerciasis and arboviruses (Table 1). The dipterans include the mosquitoes (Culicidae) as well as blackflies (Simuliidae), sandflies (Phlebotominae), tsetse flies (Glossinidae) and gnats (e.g., Sciaridae). Mosquitoes belonging to the genera *Anopheles*, *Aedes* and *Culex* transmit many aetiologic agents of diseases, including malaria, and the arboviruses, namely, yellow fever, dengue, Zika, chikungunya, West Nile fever, Japanese encephalitis and filarial nematodes (Table 1).

Mosquitoes alone kill ca. 400,000 each year, with deaths due to malaria having occurred for many decades, with deprived children < 5 years old particularly vulnerable [38,39]. Mosquitoes also transmit dengue which is a widespread viral disease, transmitted by *Aedes aegypti* and *Aedes albopictus*, and 3.9 billion people are at risk of infection each year in 129 countries, resulting in about 40,000 deaths [38,40]. Sandflies are vectors of *Leishmania* transmitted in Africa, the Americas (Brazil), the Middle East, South Asia and the Mediterranean. Leishmaniasis, together with onchocerciasis and filariasis transmitted by blackflies and mosquitoes, respectively, result in permanent disfigurement in infected people. In Africa, tsetse flies transmit *Trypanosoma brucei rhodesiense* and *T. b. gambiense*, causing sleeping sickness in those infected (Table 1). The economic burden and human suffering caused by these diseases are enormous [41].

Other important diseases transmitted by dipterans include lymphatic filariasis and river blindness (onchocerciasis) transmitted by mosquitoes and blackflies, respectively (Table 1). Filarial worms are nematodes transmitted by mosquitoes and include *Wuchereria bancrofti*, *Brugia malayi*, *Brugia timori* and *Dirofilaria* spp. Infection occurs when parasite larvae are deposited in the host following biting by mosquitoes transmitting the parasites. The parasites penetrate into the lymphatic vessels to become adults and result in inflammation and tissue damage leading to elephantiasis and other symptoms [42]. In 2000, the WHO began a “Global Programme to Eliminate Lymphatic Filariasis” which has reduced the need to use preventative chemotherapy for 740 million people [42]. Blackflies, *Simulium* spp., transmit onchcerciasis caused by the filarial nematode *Onchocerca volvulus.* When infected blackflies bite humans, they transmit parasite microfilariae and the resulting adults reside under the skin in nodules. The adults produce masses of microfilaiae which migrate through the body and can infect the eyes and optic nerves, leading to blindness [43]. Great progress is being made in controlling and eliminating this dreadful disease in many countries such as Columbia, Ecuador, Mexico, Uganda and Sudan [44].

Hemipterans infect fewer people than dipterans and result in a reduced disease impact with the parasites transmitted. Triatominae, including *Rhodnius prolixus* and *Triatoma infestans*, transmit the flagellate protozoan *Trypanosoma cruzi*, resulting in Chagas disease throughout Latin America and more recently the USA (Table 1). The disease pathology includes chronic inflammation of the heart, colon and nervous system. Of the ca. 6 million people infected with *T. cruzi*, about one third will die [45].

Fleas and lice (Table 1) have been particularly devastating insect vectors in the past. Plague, transmitted by rat fleas, has resulted in the most calamitous pandemics of mankind. “The Black Death” occurred over centuries in China and then swept through Europe leading to the Great Plague of London from 1665–1666 [46]. The bacteria immobilise human innate immunity, leading to multi-organ failure, lung infections and death. Foci of plague still remain throughout the world with recent outbreaks recorded [47]. Lice have also been associated with humans for thousands of years and epidemic typhus transmitted by the body louse, *Pediculus humanus*, resulted in some of the worst pandemics recorded. Nowadays, body lice are mainly associated with allergic reactions in deprived populations although modern wars still pose a risk for the re-emergence of epidemic typhus [48].

Unfortunately, global warming has increased the emergence and/or spread of insect-vector-borne parasitic/pathogenic diseases. In 1990, for example, the highly invasive Asian tiger mosquito, *Aedes albopictus*, was probably introduced into Europe via Albania and Italy with imported vehicle tyres. The climate was suitable and the mosquitoes spread and transmitted chikungunya and dengue imported into Europe by international travellers [49]. The rapid spread of emerging pathogens can also be explosive, as with the Zika virus pandemic in Brazil in which the virus first appeared in 2015 but by 2016 had infected ca. 211,700 people [50].

The “incredible” aspect of insect immunity, which embraces vector species too, is derived from studies of non-self recognition processes involved in insect interactions with parasites and pathogens. In particular, work with *Drosophila* showed that the insect Toll transmembrane receptor was involved in antifungal defence [51], and this led to the discovery of mammalian Toll homologues [52]. Thus, immune recognition and signaling processes in insects have close parallels with innate immune reactions in humans, and this is leading to innovative human disease therapies. For example, the field of human adjuvants for the production of vaccines is being revolutionised by the discovery of Toll-like receptors in humans with implications not only in vaccine production but also in immuno-therapies for many diseases including Alzheimer’s, allergies, cancer and drug addiction [53]. Who would have imagined that diminutive *Drosophila* fruit flies could be the source of major new information on immune functioning and innovative therapies in human diseases including COVID-19 [54]? This also emphasises the wisdom of using *Drosophila* and developing new insect-based models, such as *Galleria*, *Sarcophaga*, *Manduca* and *Rhodnius*, as powerful tools for dissecting out many aspects of the human immune responses [55].

Finally, the incredible complexity of vector–parasite/pathogen interactions must be emphasised and “*The Biology of Blood-Sucking in Insects*” [56] is still highly recommended for more background details of this. For example, variations in immune reactivity can occur with the same parasite species in different members of the same host population [57]. There are also great variations in immunity in the same host species exposed to different pathogens [58], probably related, in part, to co-infections.

**Figure 1:**
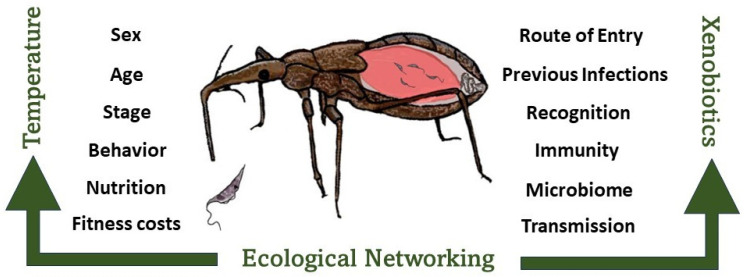
Some determinants of infectivity for parasites and insect vectors.

There are numerous determinants of successful infection or parasitisation (Figure 1). These include resistance to environmental stressors such as changes in the ambient temperature and exposure to xenobiotics, as well as previous invasions by parasites/pathogens [59]. The extent of networking occurring with other species in complex ecological communities will also affect host finding, biting and disease transmission [36]. Other determinants include the sex and developmental stage of the vector [60,61], as well as physiological factors such as the host nutrition [62] and the fitness costs of immunity [63]. In addition, the route of entry and survival in the host will also dictate the nature of the immune response [64]. The immune process itself against the invader is also not simple but, as shown in mosquitoes, composed of a tripartite response involving interplay between the gut microbiome, immune defence reactions and invading parasites [18,29]. Recently too, evidence is accumulating in insects for the presence of immune priming whereby an initial low-dose infection provides protection against a subsequent potentially lethal dose [65]. This could partially explain variations in immune capability within a population composed of insects of different ages.

## 3. Typical Insect Immune Scenario

To understand the dynamics of vector immunity to invading parasites and pathogens it is essential to understand the initial processes involved in gaining access to particular insect host tissues. Different invaders adopt different strategies for entry although routes are mainly limited to the outer cuticle and various epithelia lining the cuticle, gut, reproductive system and tracheae. Most human parasites/pathogens are ingested during blood feeding by vectors with biting mouthparts, but the subsequent events in the host vary significantly. Thus, African trypanosomes, *T. cruzi*, *Leishmania* spp. and *Yersinia pestis* remain and develop within the confines of the midgut, while *Plasmodium* spp., *Trypanosoma rangeli*, arboviruses and human parasitic nematodes migrate out of the gut and colonise and develop in other vector organs [66]. Problems of parasite/pathogen survival due to physiological incompatibilities and robust host innate immune responses have to be overcome and this topic is considered in more detail in other papers in this Special Issue. One example of the effect of these problems is illustrated by African trypanosomes in which, in some host/parasite combinations, less than 1% of parasites survive in the tsetse fly vector by 3 days post-infection [67].

The basic insect immune response can be elicited in response to foreign invasion following wounding or in the gut, the haemocoel, the haemocytes, the fat body, the salivary glands and other tissues following ingestion. Recognition is mediated by means of pattern recognition receptors (PRRs) located on/in immune tissues which bind to pathogen-associated molecular patterns (PAMPS) of invading parasites and microbes. This recognition and binding can directly activate innate immune responses like phagocytosis and also initiate various signaling pathways leading to transcription of immune effector genes and the secretion of immune proteins/cytokines for eliminating invaders [68]. The insect effector immune factors include antibacterial peptides (AMPs), lectins, prophenoloxidases (PPOs, for melanisation), reactive oxygen species (ROS), nitric oxide (NO), antiviral factors, cytokines and many more [17,68,69]. Much of the knowledge of these processes was gleaned by work on *Drosophila* [70,71,72] while valuable contributions have also been made with other species including *Aedes/Anopheles* mosquitoes [18,29,73,74,75], *Hyalophia cecropia* [76], *Manduca* [77,78], *Galleria* [79,80,81] and *Bombyx* [82,83] moths, *Sarcophaga* fleshflies [84,85] and *Rhodnius* assassin bugs [86,87].

Insect immunity is usually described as consisting of interacting humoral and cellular elements. The humoral components, often derived from the immune tissues, involve immune proteins and pathways, mentioned above, such as the antimicrobial peptides and melanisation reaction, while cellular elements include coagulation, phagocytosis, nodule formation and encapsulation-type responses [17,68,69].

## 4. Vector Cellular Immunity

Central to many of the immune responses are the haemocytes which can be free in the circulation or sessile and associated with tissues such as the heart ostia of mosquitoes [68]. One recurring problem for many workers has been the extraction and identification of the types of haemocytes present. Previous work on haemocyte classification [88,89,90] is still helpful in overcoming these problems. Some haemocyte types are extremely fragile, including those from vector species, and usually degranulate and break down rapidly on exposure to air. The use of an anticoagulant solution stabilises the cells and allows examination under phase optics to observe both the structure and some functions of these cells. In addition, with an anticoagulant it has been possible to separate *Galleria mellonella* cell types [91], study their interactions in vitro [92] and stain specific cell types with monoclonal antibodies [93]. Smearing the insect blood and treating with Giemsa stain, as reported constantly, is not recommended.

### 4.1. Haemocyte Types

The huge diversity of insects is also reflected in variations in the form of the different types of haemocytes present, although the use of phase observations of stabilised cells can distinguish which cells are phagocytic or involved in coagulation reactions due to granule discharge. Studies on 15 insect orders identified the main haemocyte types as prohaemocytes, plasmatocytes, granular cells, oenocytoids, spherule cells and coagulocytes, with the number of these classes present varying with different insect species and during various stages of development [89]. The recent ability to study the function of single haemocytes [16,94,95,96,97], using single-cell RNA sequencing (scRNA-seq) combined with gene silencing and functional studies, is a breakthrough in typing these cells. In *Drosophila*, scRNA-seq has identified many subpopulations of plasmatocytes at different stages of development [94,95], while in mosquitoes immune cell subsets, their differentiation and lineages have also been defined [16,96,97].

Haemocytes in vectors have mainly been studied in mosquitoes and triatomines. In mosquitoes, three main cell types, namely, prohaemocytes, granulocytes and oenocytoids, have previously been described (Table 2), but the study of haemocyte classification, as mentioned above, has been revolutionised by RNA-seq and other modern molecular techniques. Thus, subsequently, in mosquitoes, the haemocytes identified are subtypes of these three haemocyte classes. Kwon et al. [97] identified seven haemocyte subtypes in *Anopheles gambiae* with four different populations of granulocytes, two populations of oenocytoids and the prohaemocytes. These populations were identified using scRNA-seq analysis combined with homology studies, phagocytosis and phagocyte-depletion assays. Severo et al. [96] and Raddi et al. [16] used similar methods in classifying mosquito haemocyte subtypes. Severo et al. [96] only detected two subtypes, granulocytes and oenocytoids, while the results of Raddi et al. [16] complemented those of Kwon et al. [97], with the addition of another cell type designated as megacytes.

We have now entered a new era with the use of advanced molecular, labelling and functional techniques. In addition, detailed analysis of haemocyte ontogeny is available showing the functional plasticity of subpopulations of previously statically classified haemocyte types. With these new techniques great care must be taken to ensure that the perfused haemocytes remain stable, especially in manipulations involving labelling and cell-counting techniques. In addition, checks should be made to confirm that perfusion removes any large populations of sessile haemocytes attached to various organs in the body. Examination of the methods of some recent papers clearly indicates that the haemocytes are often perfused with non-sterile solutions, magnetic beads, etc. and placed for many minutes to attach on slides, and then treated with various antibodies or non-sterile probes. Even if perfusion is carried out with anticoagulant solutions, some haemocyte types will still react with any PAMPs in solutions and the environment to discharge their contents and elicit coagulation-like responses. The cells also often break down after degranulation with attachment to or ingestion by other cells. This will likely result in errors in identification of cell types and shows a basic lack of understanding of the fragility of some insect immune cells. The literature is full of accounts of the rapid discharge and breakdown of insect cell types on exposure to environmental factors like the PAMPs. One source of PAMPs such as endotoxin could be the water from stills and deionising columns which may not fully remove endotoxin [98]. Endotoxin from Gram-negative bacteria can activate mammalian leukocytes at concentrations as low as 0.01 ng/mL. Anticoagulant solutions also reduce the viability and attachment of haemocytes in other arthropod immune systems [99].

**Table 2:** Haemocyte types * in vector insects mainly based on morphological and functional studies.

	Haemocyte Types	Prohaemocytes	Plasmatocytes	Granulocytes	Oenocytoids/ Crystal Cells?	Cystocytes	Adipohaemocytes	Others
Vectors								
Mosquitoes ^1^		+	+/−	+	+	−	−	−
Blackflies ^2^		+	+	+	+	−	−	+
Sandflies ^3^		+	+	+	+	−	+	−
Tsetse flies ^4^		+	+	+	+	−	+	+
Lice ^5^		−	+/−	+/−	−	−	−	−
Fleas ^6^		−	+/−	+/−	−	−	−	−
Triatomines ^7^		+	+	+	+	+	+	+

* The papers cited are mainly those with some identification of the types of haemocytes studied. ^1.^ **Mosquitoes**, Hall [100]; Castillo et al. [101]; Hyllier and Strand [102]; Kown et al. [97] used RNA-seq analysis and showed 7 subpopulations of haemocytes in *An. gambiae.* ^2.^ **Blackflies**, Silva et al. [103]; Luckhart et al. [104] also described spherulocytes; Cupp et al. [105] reported small and large granule cells. ^3.^ **Sandflies**, De Albuquerque et al. [106]. ^4.^ **Tsetse flies**, Matetovici et al. [12] record melanin-producing crystal cells; East et al. [107] and Kaaya et al. [108] described spindle cells and thrombocytoids too. ^5.^ **Lice**, Coulaud et al. [109] described only phagocytes which are likely to be plasmatocytes or granulocytes. ^6.^ **Fleas**, Kozlov et al. [110]; Munoz et al. [111] described only phagocytes which are likely to be plasmatocytes or granulocytes. ^7.^ **Triatomines**, Azambuja et al. [112]; Moyetta et al. [113]. The granular cells were often giant cells and maybe ingested other cells. +/− indicates uncertainty in identification of cell type.

The haemocytes of triatomines have been described in detail by some authors although there can be some variations from species to species and according to the preparation technique used [112]. In most studies, prohaemocytes, plasmatocytes, granular cells, oenocytoids and adipohaemocytes are recognised [112,113] with giant cells and cystocytes also identified in *R. prolixus* and *Dipetalogaster maxima* [112,113] (Table 2). The more recent study of the haemocytes of *D. maxima* also used immunofluorescence and flow cytometry analysis and seemed to confirm the classification of cell types described above. Results of research on the haemocytes of triatomines using using scRNA-seq analysis combined with homology studies, phagocytosis and phagocyte-depletion assays are awaited, as utilised for *Drosophila* and mosquitoes [16,94,95,96,97].

Regarding blackflies, different species of blackflies are reported to have four types of haemocytes although these varied with the species studied (Table 2). Thus, Luckhart et al. [104] described prohaemocytes, plasmatocytes, granulocytes and spherulocytes in *Simulium vittatum*, while Silva et al. [103] described a similar profile except oenocytoids occurred instead of spherulocytes in *Ectemnaspis rorotaense* and *Ectemnaspis trombetense.* For these studies, an anticoagulant solution was perfused before harvesting the haemolymph and placing on slides for air drying before staining; a process very likely inducing haemocyte degranulation and lysis, as described above.

Studies on sandfly haemocytes are wanting except for that of Albuquerque [106] on *Lutzomyia migonei* for which five haemocyte types were identified, namely, prohaemocytes, plasmatocytes, granulocytes, adipohaemocytes and “eonocites” (oenocytoids?) (Table 2). The insects were perfused with anticoagulant and slides air dried and stained with Giemsa, so this is yet another example of possible loss of cell types. Anticoagulant is not a fixative and haemocytes can attach to slides in such solutions and, combined with air drying, change dramatically.

Tsetse fly haemocytes have been described by East et al. [107] and Kaaya et al. [108]. These are prohaemocytes, plasmatocytes, granulocytes and adipohaemocytes in all stages, as well as thrombocytoids and spindle cells in adults (Table 2). These papers were published before the introduction of anticoagulants. Despite the apparent lack of oenocytoids, the haemolymph of tsetse flies does melanise (see Section 4.2 *Cellular Defence Mechanisms*, below), and subsequently melanin-producing crystal cells have been recorded (equivalent to oenocytoids of other insects) by Matetovici et al. [12].

Regarding lice, little is known about haemocyte types, all of which are described as phagocytes by Coulaud et al. [109] who collected the haemolymph using an insulin syringe and then added this to various culture media obviously under sterile conditions. Cells survived well and phagocytosed microbes but probably did not include all haemocyte types present in vivo. In fleas, Kozlov et al. [110] identified proleukocytes, leukocytes, trophic cells and oenocytoids while Munoz et al. [111] termed all flea haemocytes as phagocytes and did not distinguish cell types.

### 4.2. Cellular Defence Mechanisms

These include coagulation, phagocytosis, nodule formation/encapsulation, apoptosis, autophagy and extracellular traps. Dividing immunity into cellular and humoral reactions is often arbitrary as these components frequently interact in most immune responses.

#### 4.2.1. Coagulation

Coagulation of insect haemolymph occurs in most studies of different species and is an essential process limiting the excess loss of haemolymph and preventing the entry of parasites and pathogens. Haemolymph coagulation has been reported in mosquitoes [114], triatomines [112,113], blackflies [104], tsetse flies [12] and lice [115]. It basically occurs in two phases, initially involving the cross-linking of clot components from the plasma and haemocytes and depending on transaminase activity followed by phenoloxidase hardening and melanising the soft clot material [116]. In mosquitoes, the process involves aplolipophorin-1 and phenoloxidase with the latter more important than in *Drosophila* [114]. Recent studies on haemolymph coagulation in vector insects are very limited and in blackflies not mentioned directly but can be interpreted in the figures published [104]. In addition, in lice, the possibility of haemolymph coagulation is inferred by transcription studies detecting the humoral immune-related genes hemocytin and noduler [115], since these are associated with sticky fibrous structures exocytosed from granulocytes and nodule formation in other insects [117]. The presence of haemolymph clotting in fleas has also been implied [111].

#### 4.2.2. Phagocytosis

Phagocytosis by insect haemocytes has been widely reported as an important defence process in vector insects. Detailed studies of the interaction of the haemocyte cell surface and free pattern recognition receptors (PRRs) with the pathogen-associated molecular patterns (PAMPs), as well as the role of plasma opsonins, have mainly been confined to mosquitoes. The comprehensive studies published on the immune system of the mutants of *D. melanogaster* provide useful guides for comparison with mosquitoes as does RNA interference (RNAi) utilising the multiple mosquito genome sequences available [118]. Some modest progress, however, has recently been made in understanding haemocyte-mediated phagocytosis in other vector insects [104,105,110,111,115,119].

Details of mosquito phagocytic interactions with parasites and pathogens are given in other papers in this Special Issue and have been published previously [15,102,118,120,121,122,123,124,125]. Granulocytes are the main phagocytes in mosquitoes and comprise about 90% of the haemogram of which there are ca. 2000–5000 cells [126]. These haemocytes are either free in circulation or form sessile concentrations, the periosteal cells, around the heart valves (ostia) in adults, and in tracheal tuffs in larvae [121]. Both free and sessile haemocytes rapidly phagocytose small particulates including viruses, bacteria, fungal elements and protozoan parasites.

The recognition and phagocytosis of invading pathogens and parasites depend upon a variety of putative PRRs (more details of recognition are given in Section 6 on recognition, below). These include the thioester-containing proteins (TEPs), identified in *D. melanogaster*, *An. gambiae* and *Aedes aegypti* and which are mostly free in the haemolymph. One of these, TEP1 in mosquitoes, is secreted into the haemolymph and enhances phagocytosis following its activation by LRIM1 and APL1C to form a complex binding to and destroying bacteria and *Plasmodium* ookinetes [122]. Numerous other components of the mosquito immune system, including families of proteins, such as fibrinogen-related proteins (FREPs), C-type lectins and Gram-negative binding proteins (GNBPs), as well as the signaling pathways, have been characterised in mosquitoes and influence the activity of the immune response to parasite/pathogen invasion [122] (see Section 5 Vector Humoral Immunity, below).

A fascinating study on the mosquito responses to *Plasmodium* has shown that infection results in recruitment of additional periosteal haemocytes to amplify the heart-related immune response. This results from the upregulation of the IMD and JNK signaling pathways (see Section 6 on recognition, below) which enhances this periosteal haemocyte aggregation, activating phagocytosis and melanisation in the heart, thereby demonstrating the integration of the response between the immune and circulatory systems [125]. The freely circulating haemocytes were not affected in a similar way. Finally, recent work with dengue- and Zika-infected *Ae. aegypti* mosquitoes showed that phagocytosis by haemocytes is not needed for controlling viral infection in the midgut but is vital for restricting systemic viral dissemination [124].

Information on phagocytosis in other vector insects has been published, although the details available are often limited and may be related to whether the invading parasites/pathogens are confined to the gut or invade other tissues in the body.

- In blackflies, phagocytosis of erythrocytes and bacteria is mediated by plasmatocytes and granulocytes with evidence of these cells also being involved in remodelling tissues [104].
- With sandflies, *Leishmania* development occurs exclusively in the gut with research mainly confined to this organ and interaction with the microbiota [8], so that consideration of the possible role of the haemocytes has been neglected. *Lutzomyia longipalpis* embryonic cell lines used for studying innate immunity in sandflies, however, have active Toll and Imd pathways and interaction with *Leishmania* parasites was reported [127], so the haemocytes may well be activated following infection.
- In lice and fleas, more information on phagocytosis has been published recently.
  In head and body lice, the relative phagocytic activities of the haemocytes have been compared following injections of *Escherichia coli* or *Staphylococcus aureus* and showed that the body lice had a reduced immune response compared to the head lice [115]. In addition, haemocytes have been identified engulfing endosymbionts during their migrations around the body of the lice [128]. The reduced phagocytic competence of the body lice may be related to the increased pathogen-vectoring capacity of these insects [128]. The presence in lice of the genes for the main signaling pathways, except Imd, may indicate the potential for activation of the haemocytes [129].
- The phagocytic activity of the haemocytes of fleas has been the subject of similar research to that in lice [111], so that following inoculations of *E. coli* the phagocytic activity of the haemocytes increased significantly. This was accompanied by a general enhancement of antimicrobial resistance of the haemolymph, probably also involving humoral immune factors induced via signaling pathways [130] (see Section 5 Vector Humoral Immunity, below).
- Two other major vectors in which phagocytosis has been recorded are the tsetse flies, *Glossina* spp., and the triatomines, *Rhodnius* and *Triatoma.* The main parasites involved in these insects are the African trypanosomes and *T. cruzi*, respectively. These parasites are mainly confined to the vector gut, although African trypanosomes do migrate in the vector during maturation [12], and *Rhodnius* also hosts *T. rangeli* which invades the haemocoel [131]. The tsetse fly association with the invading African trypanosome is extremely complex since the phagocytic ability of the haemocytes against bacteria has been recorded [132], as has a specific genomic expansion within the thioester-containing protein (TEP) family [133] which is usually regarded as opsonic for phagocytosis.

In a series of fascinating experiments, comparing tsetse fly survival following *E. coli* challenge of antibiotic-treated, aposymbiotic tsetse flies with wild-type, flies transfused with either wild-type fly haemolymph separated into its cellular or soluble fractions showed that all those flies receiving the soluble fraction died by 12 days while 62% of those with the cellular fraction survived. In this innovative study, much additional evidence confirmed the importance of cellular immunity in tsetse defences against foreign invaders [132]. The importance of this study with tsetse flies (6–16 mm length) should not be overlooked as the ability to repeat such experiments with smaller vector insects such as sandflies (ca. 3 mm), fleas (1–3.2 mm), lice (0.5–5 mm), blackflies (2–6 mm) or mosquitoes (3–6 mm) would be challenging.

The immune reaction of the tsetse flies, however, to trypanosome infection also includes other factors, such as peptidoglycan recognition proteins (PGRPs), antimicrobial peptides (AMPs), a tsetse EP-protein, interactions with the Imd pathway and the role of the symbiont, *Wigglesworthia*, in immune efficacy [12,134] (see Section 5 Vector Humoral Immunity, below, and in other papers in this Special Issue).

Most research on triatomines has been carried out on *R. prolixus* and considerable progress in understanding the complex interaction of *T. cruzi* with the insect vector is being made [4,24,135,136,137,138,139]. Phagocytosis by the haemocytes in *Rhodnius* has been described following *T. rangeli* invasion from the gut into the haemolymph and following injection of bacteria into the haemolymph [140]. However, what is particularly interesting are experiments showing the induction in the haemolymph of an early systemic immune response following the colonisation by *T. cruzi* of the vector gut. Therefore, communication of parasite invasion is transmitted rapidly to the innate immune system following oral infection [24]. This, of course, has implications for many other vector–parasite associations in which the parasites remain confined in the gut (see Section 5.7 *Triatomines*, below).

#### 4.2.3. Nodules and Capsules

Similar to phagocytosis, nodule formation and encapsulation (often referred to together as capsules) occur rapidly following invasion of a range of insects by parasites and pathogens. The encapsulation response may be humoral and melanotic, as in some dipterans with limited numbers of haemocytes, solely cellular as in lepidopterans like *Galleria* or even formed by a combination of humoral and cellular responses [141].

The formation of these structures is controlled by interacting cytokines and effector molecules, details of which have been identified mainly in *Drosophila*, lepidopterans and non-vector insects [17,72,142,143]. Nodules/capsules have been described as forming as a result of insect invasion by large numbers of microbes or by parasites too large to be ingested by a single haemocyte [141]. Recently, however, it has been shown that the initial haemocyte aggregation process, typical of capsules, can occur following contact with one or a small number of labelled yeast cells [142]. During this process, granule-containing cells stick together due to their release of a viscous material identified as hemocytin which is a homologue of the mammalian von Willebrand factor [142,143]. The degranulation of the haemocytes occurs after recognition of PAMPs by PRRs, activation of a haemolymph serine proteinase and Spätzle1, and with Toll signalling releasing 5 -HT and eicosanoids to aggregate the cells [142]. In addition, although good progress has been made in understanding aspects of phagocytosis and capsule formation in model insects, like *Drosophila*, *Galleria* and *Bombyx*, how exactly these early events trigger subsequent stages in capsule formation and humoral responses from the fat body and other tissue needs clarification. The work of Sato [142] discusses some of these issues in detail (see Section 5.7 *Triatomines*, below).

Regarding capsule formation in vector insects, details of this process are mainly confined to mosquitoes and triatomines. In mosquitoes, the invasion by *Plasmodium* and nematodes results in encapsulation-type responses surrounding the parasites. In refractory mosquitoes, the ookinetes become surrounded by a capsule which melanises and the parasites are lysed by a complement-related thioester-containing protein (TEP1) [144]. The capsule is composed of eumelanin and pheomelanin with 14 host proteins embedded including *Ag*Mesh with domains indicating a recognition function [145]. This is interesting as perhaps the capsule is not just an inert structure walling off lysed ookinetes but during formation may communicate with the immune system and activate a host response.

There have also been numerous studies on the encapsulation of filarial nematodes in mosquitoes, since these parasites include medically important species resulting in lymphatic filariasis, such as *Wuchereria bancrofti*, causing 90% of human cases, *Brugia malayi* and *Brugia timori.* These are transmitted mainly by *Culex* in urban regions, *Anopheles* in rural areas and by *Aedes* in islands of the Pacific [42]. Another filarial nematode, *Onchocerca volvulus*, is transmitted by *Simulium* spp. blackflies and causes river blindness.

The nematode microfilariae are ingested by the insect vectors during feeding on the blood of mammalian hosts and then penetrate the insect midgut epithelium to enter the haemolymph. They subsequently migrate to the thoracic musculature, invading the flight muscles before moulting twice into third-stage infective larvae. These migrate to the head to reach the proboscis where they can infect another human host during the wounding process of a blood meal [146]. It is during the migration of the microfilariae around the body in the haemolymph that the parasites encounter the cellular and humoral encapsulation reactions of the vector. These reactions include melanotic encapsulation of the microfilariae, often involving an initial deposition of melanin granules and cellular debris on the parasite surface, followed by plasmatocyte attachment in later stages [147].

The importance of melanin in mosquito defence against invading microfilariae may be indicated by the RNAi knockdown of phenylalanine hydroxylase (a melanin precursor) in *Ae. Aegypti* and *Armigeres subalbatus*, resulting in the reduced melanisation of invading *Dirofilaria immitis* microfilariae [148]. There are also many other studies confirming the important role of melanin and its precursors phenoloxidase (PO) enzymes in insect immunity. For example, in *Drosophila*, the genome encodes three precursor POs, designated as PPO1, PPO2 and PPO3, and flies were generated with deletions for PPO1 and/or PPO2. Analysis of these mutants alone and in combination identified the functions of both PPO1 and PPO2, but not PPO3, in melanisation of the haemolymph and in encapsulation of parasitoid eggs. This study also showed an essential role of melanisation in defence against some Gram-positive bacteria and fungi [149]. In addition, *Ae. aegypti* has 10 PPO genes, 4 of which are transcriptionally activated by Cactus silencing which also leads to the arrested development and death of *Plasmodium gallinaceum*. This research indicates that the PPO gene expression and its RUNT-related transcription factor 4 (RUNX4) are controlled by the Toll pathway (see Section 6 on recognition, below) and are important in restricting parasite development [150]. Here, we have included the encapsulation of nematodes in mosquitoes within Section 4.2 *Cellular Defence Mechanisms.* In fact, these capsules probably are generated by interactions between the cellular and humoral immune defences of the mosquito.

Recent work has revealed the true complexity of the mosquito immune response to nematode invasion. For example, very useful studies on mosquito vectors during the first 12 h of infection have compared the transcriptomic profiles of resistant and susceptible *Ae. aegypti* to *B. malayi* filarial nematodes. Differentially regulated genes were found including those involved in antimicrobial peptides, recognition proteins including lectins, signaling components of the Imd, Toll and JAK/STAT pathways and serine proteases [151]. Other immune-related factors such as the generation of reactive oxygen and nitrogen radicals, the influence of symbiotic bacteria like *Wolbachia* on immunity, the role of parasite extracellular vesicles and avoidance mechanisms of the host response also need consideration [17,152,153,154]. Some of these are mentioned in Section 5 Vector Humoral Immunity, below.

In triatomines, *T. cruzi* and *T. rangeli* form aggregates in the anterior midgut and in the haemolymph, respectively, of *R. prolixus.* The *T. cruzi* aggregates are formed entirely of parasites which develop confined to the gut and result from lectin present in the gut and may serve to protect the parasites from the lytic activity present in this organ [155]. True melanising nodules consisting of parasites and haemocytes are only formed after invasion of *T. rangeli* from the gut into the haemocoel or by injection of bacteria [135]. In *R. prolixus* haemolymph, a galactose-binding lectin enhances nodule formation in vitro by *T. rangeli* [156]. Subsequently, this molecule was identified as a rhamnose-binding lectin with potential importance in the host defence of *Rhodnius* against *T. cruzi* and *T. rangeli* infections [157]. Although certain strains of *T. rangeli* appear to be broken down within nodules, others survive and multiply, modulating the host response [135]. This process may involve the effects of eicosanoids and platelet-activating factor (PAF) on the PPO system and haemocyte aggregation [135]. More recently, a quantitative proteomics study of *T. cruzi* analysing the expression profiles of *Rhodnius* haemolymph proteins from 6 h to 24 h post-infection identified 12 novel immune proteins of unknown functions [24]. A similar approach following early *T. rangeli* infection would probably be just as rewarding and identify the differential expression of haemolymph proteins in response to *T. rangeli* compared with *T. cruzi* (see the detailed paper by Schaub et al. on triatomines in this Special Issue [138]).

In *Glossina*, there are reports of encapsulation-type reactions towards injected bacteria or implants [12,132,158] with few details given except that the reaction appears to just involve a few haemocytes and with melanin deposition occurring.

Apart from the classical haemocyte defence reactions against invading parasites, described above for insect vectors, more recently, additional immune responses have been reported. These responses include RNA interference (RNAi), apoptosis, autophagy and extracellular traps [159,160]. RNAi, apoptosis and autophagy occur in response to viral infections whilst extracellular traps in *Rhodnius* deal with a range of microbes [159,160]. These reactions are not confined to haemocytes but more generally involve other tissues in the body such as the intestine, Malpighian tubules, fat body and salivary glands. More information on some of these is available in the above references as well as in Section 6 on recognition.

## 5. Vector Humoral Immunity

Table 3 shows a summary of some of the more important aspects of the humoral immune factors reported in vector insects. These factors are conservative throughout the insects but are expressed and utilised to a greater or lesser extent from species to species and even within species. The majority of the papers published on vector immune responses to parasites and pathogens are those dealing with humoral responses in mosquitoes, due to their transmission of malaria, with triatomines in second place a long way behind. Most of the remaining vector insects have received scant attention and published studies are mainly confined to humoral immunity since the vectors are either very small and contain few haemocytes (e.g., sandflies) or else the parasites transmitted do not invade the haemocoel (e.g., sandflies, tsetse flies). One important aspect of the vector-insect–parasite/pathogen interactions, rapidly becoming more evident, is the complexity of these associations so that only basic information can be summarised here with the reader referred to the additional papers in this Special Issue for details.

In contrast to Section 4, in which the main cellular immune processes were described separately, here the humoral immunity of each vector group is briefly described. This allows a more dynamic and interactive summary of this complex process.

### 5.1. Mosquitoes (Table 1 and Table 3)

The sequencing of the genomes of *An. gambiae*, *Ae. aegypti* and *Culex quinquefasciatus* [188,189,190] provided great opportunities for new approaches for studying the biology of these important vectors and has led to rapid progress in understanding the vector–parasite/pathogen associations. *Aedes aegypti* is responsible for vectoring yellow fever, dengue, Zika and chikungunya and filarial nematodes, *An. gambiae* for malaria and filarial worms and *C. quinquefasciatus* for West Nile and Saint Louis encephalitis viruses and the filarial worms themselves (Table 1).

The mosquito responses to these diverse pathogens may well vary with new PAMPs to be recognised on/in these, as well as with the different vector species whose immune competence is affected by the composition of the microbiota [18,191]. For example, in *Aedes*, *Anopheles* and *Culex*, 417, 380 and 500 immunity genes have been recorded, respectively, with expansions in *Culex* of C-type lectins, fibrinogen-related proteins (FREPs) and serine protease inhibitors (SRPNs) accounting for some of the increases in immunity gene numbers [161].

Following ingestion with the blood meal, the pathogens enter the midgut where they face numerous host-derived factors which may inhibit/kill the invaders or else assist in their development. The epithelial cells of the midgut, however, are protected by a chitinous/glycoprotein peritrophic membrane, although in *An. gambiae*, FREP1 anchors *Plasmodium* to the peritrophic matrix and assists parasite penetration of this structure [162]. Regarding arboviruses, commensal bacteria may assist the infection process in the mosquito midgut [192], while disrupting the formation of the peritrophic membrane by RNAi of chitin synthase expression has no effect on *B. pahangi* development or on the spread of dengue virus [193].

Once in the midgut, many factors are involved in the survival or elimination of the parasite, including:

- upregulation of immune effector genes towards antimicrobial peptides (AMPs) via signalling pathways;
- the vector PPO system, melanisation and serine proteases;
- cytotoxic/stimulatory lectin molecules;
- nitric oxide and ROS killing of parasites;
- specific peptides stimulating parasite differentiation;
- glycoprotein receptors on the surface of the midgut for parasite attachment;
- role of bacterial symbionts.

Recognition by the PRRs of the PAMPs of invading parasites and pathogens occurs shortly after entry of the blood meal into the gut and other tissues of vectors and results in the systemic upregulation of genes and immune effector molecules [24]. These are produced by triggering of the three signalling pathways, Toll, Imd and JAK-STAT, and details of these are given in Section 6 on recognition, below, and in other papers in this Special Issue.

i. Important groups of effector molecules are the antimicrobial peptides against bacteria, fungi, *Plasmodium*, viruses and nematodes [24,126,151]. Four important AMPs in mosquitoes are defensins, cecropins, attacins and gambicins (Table 3). An example of the AMPs’ role against invaders is provided by RNAi silencing in *Ae. aegypti* of the Toll factor, Cactus, resulting in enhanced expression of the defensin gene and helping to control/neutralise dengue virus. Some bacterial species in the gut can also promote the expression of AMPs as with *Serratia marcescens* in *An. stephensi* against *Plasmodium berghei* [194]. In mosquitoes, it is not clear which pathway induces which AMP but some are modulated by both Toll and Imd and this may indicate that these two signaling pathways partially converge downstream in response to infection with different pathogens [19]. The role mosquito AMPs play against bacteria, fungi, *Plasmodium* and viruses is recorded but interaction with nematodes is not fully understood, although previous priming of *Ae. aegypti* exposed to *B. malayi* significantly reduces infection intensity [195].

ii/iii. The vector PPO, melanisation, serine proteases and C-type lectins (CTLs) are all involved in the mosquito immune defences against pathogens (Table 3). Melanisation of nematode microfilariae and *Plasmodium* ookinetes is described above in Section 4.2.3 Nodules and Capsules and the mosquito haemocytes have been shown to be significantly involved [102]. There are, however, reports of melanisation of nematode larvae in the midgut before entry into the haemolymph [196] and other confirmations that the interaction of this key defence process with pathogens is not fully understood in mosquitoes [197]. For example, in *Ae. aegypti*, the filarial nematode *B. malayi* has recently been shown to produce extracellular vesicles that downregulate the AAEL002590 gene encoding a serine protease involved in mosquito PO activity [152]. In addition, activation of the *An. gambiae* melanisation response is mediated by complex extracellular hierarchical cascades involving CLIP-domain serine proteases (CLIP-SPs), namely, SPCLIP1, CLIPA8 and CLIPA28 upstream of CLIPC9 [197]. To further emphasise the complexity of melanisation, the genomes of *Ae. aegypti*, *An. gambiae* and *Cx. quinquefasciatus* have 52, 55 and 25 CTLs predicted, respectively [165], and have also previously been shown to be involved in PPO activation in other insects [166,167]. In *An. gambiae*, too, CTLs play an important role in interactions with *Plasmodium falciparum* parasites with CTL4 protecting the human parasite from a killing mechanism that is independent of the normal TEP1-mediated Imd pathway melanisation process [167]. In contrast, defence against the rodent parasite *P. berghei* involves TEP1 and the Imd pathway so that the mosquito immune system has different roles depending upon the pathogen [167]. The literature, however, also shows that in *Anopheles coluzzii*, the immune genes APL1C, LRIM1, TEP1 and TEP3 and the signaling pathways influence susceptibility to both fungal and *P. falciparum* infections [198].

iv. In addition, reactive oxygen (ROS) and nitric oxide (RNS) species are also involved in mosquito killing of pathogens [122,196]. The blood meal alone results in ROS/RNS production but when *Plasmodium* passes through the midgut epithelium this is enhanced further [6]. Strains of *An. gambiae* resistant to bacteria also have higher levels of ROS and the application of antioxidants decreases mosquito survival [199]. Furthermore, *P. berghei* infection often produces high levels of mosquito killing which can be reduced by the oral application of the antioxidant uric acid [199]. It has been proposed that toxic quinones generated during melanotic encapsulation also generate high levels of ROS to kill parasites [168] and that in the mosquito midgut epithelium responses to *Plasmodium* are modulated by ROS from mitochondria [169]. Regarding RNS activity, a nitric oxide synthase (NOS) gene is transcriptionally activated in *An. gambiae* by both bacteria and *Plasmodium* parasites and is particularly active in the midgut wall where the synthesis of nitric oxide (NO) occurs by inducible NOS (AsNOS) [200,201]. Furthermore, mosquitoes fed with the NOS substrate, L-arginine, have reduced infection rates by 28%, while the NOS inhibitor, L-NAME, significantly increases oocyst numbers in the midgut wall [201]. More recent research has revealed the role of RNS in the mosquito defences against invading *Plasmodium* parasites. The invasion of *An. gambiae* (G3) midgut epithelial cells by ookinetes causes a wounding response resulting in apoptosis and also induction of NOS, haeme peroxidase (HPX2) and NADPH oxidase 5 (NOX5) enzymes in these cells. The NO produced is toxic and results in protein nitration of the midgut basal lamina which attracts haemocytes. Upon contact with the midgut nitrated surface these release haemocyte-derived microvesicles (HdMvs) which activate the mosquito (TEP1) complement system to lyse the parasites [170,171].

v/vi. There have been many reports of parasite proteins required for *Plasmodium* development and invasion of mosquitoes but fewer on the role of mosquito midgut proteins assisting parasite invasion [202]. Such proteins include *Anopheles* alanyl aminopeptidase N (AnAPN1), fibrinogen-related protein 1 (FREP1) and *An. gambiae Plasmodium falciparum* P47 receptor (AgPfs47Rec). AnAPN1 is a midgut lumen surface glycoprotein functioning to digest the blood meal and binding to ookinetes to facilitate their entry into midgut cells for continuation of sporogenesis [202,203]. FREP1, as mentioned previously, anchors *Plasmodium* to the peritrophic matrix and assists parasite penetration of this structure [162]. AgPfs47Rec is an *Anopheles* midgut receptor for *P. falciparum* protein Pfs47. This interaction mediates the parasite avoidance of mosquito immunity by disruption of the c-Jun-N-terminal kinase (JNK) signaling pathway leading to inhibition of the midgut nitration process and the TEP1 complement system [202,204].

vii. Research on the mosquito microbiome is most important since it has been shown that the component microbes have roles in the physiology, nutrition, metabolism, immunity, reproduction, longevity and behaviour of these vector insects [26,205]. The microbiome also modulates the interaction of the vector with invading parasites and pathogens [18,26,205]. The reviews by Gabrieli et al. and Vinayagam et al. [18,29] describe the trilogy of interactions between the mosquito gut microbiome, the vector immune system and invading pathogens. The blood meal in a female mosquito triggers the proliferation of the midgut microbiota whose cell wall peptidoglycan is recognised by the peptidoglycan recognition protein LC (PGRP-LC) in the anterior midgut, resulting in activation of the Imd pathway [18,29]. The activated Imd pathway eventually induces the expression of antimicrobial peptides (AMPs) via the NF-κB transcription factor Relish. This priming of mosquito immunity by the gut bacteria to express AMPs explains one way by which the microbiome defends against invading parasites and pathogens. For example, *S. marcescens* in *An. stephensi* defends against *P. berghei* [194] and *Proteus* sp. in *Ae. aegypti* protects against dengue [206].

Much work has concentrated on the bacteriomes of *Aedes* spp. and *Anopheles* although the mosquito microbiomes also contain fungi, viruses, archaea and protozoans [26,207,208]. The bacteriomes of these mosquitoes are composed mainly of Gram-negative species. In anophelines as many as 98 genera have been recorded [209], although core microbiota usually dominate [26,210]. The origin of the microbiome seems partially to depend on the ecology as different mosquito species from comparable environments have similar core bacteria [211]. The fact that these bacteria can reduce *Plasmodium* infections has stimulated interest in developing paratransgenesis (genetic manipulation of the insect vector’s native microbiome to inhibit or kill invading disease pathogens) to control mosquito-transmitted diseases, such as malaria, and maybe mosquitoes too. The main bacteria of interest include *Asaia*, *Pantoea*, *Escherichia*, *Serratia*, *Enterobacter*, *Chromobacterium* and *Pseudomonas* [26]. For example, *Asaia* strains inhibit *Plasmodium* development by producing toxic proteins [212], reduce parasite numbers by activating *Anopheles* immunity after infective feeding [213] and also inhibit competing *Wolbachia* infections [214]. Wang and Jacobs-Lorena [215] recognised four classes of anti-*Plasmodium* effector molecules: (i) parasite killers; (ii) those engaging with parasites; (iii) those engaging with epithelia of the mosquito midgut or salivary glands; and (iv) modulators of the mosquito immune system. These effector molecules with different modes of action can potentially be engineered in combination into symbiotic bacteria, such as *Pantoea agglomerans*, to kill parasites and prevent the development of resistance [215].

### 5.2. Blackflies (Table 1 and Table 3)

There are more than 2200 species of blackflies of which the largest genus, *Simulium*, has at least 26 species that are vectors of *Onchocerca volvulus* [216]. Many recent papers on this important group are concerned with “prevention, control and elimination” [43,44] together with taxonomy, infection rates and composition of the saliva e.g., [172,217]. Unfortunately, since the pioneering research of Ham, Hagen et al. in the 1990s and early 2000s [173,174], there have been few papers on the details of the interaction of the blackfly immune system in the midgut and haemolymph with the microfilariae following parasitisation by *O. volvulus*. Therefore, vaccine development against *O. volvulus* has concentrated on antigens associated with the parasites rather than on elements of the vector immune response [43].

The usual comment about the life cycle of the microfilariae in the blackfly vector is that following an infected blood meal the parasites “develop further in the black fly and are then transmitted to the next human host” [44]. In fact, Ham et al. [173] described four types of molecules modulated by ingested microfilariae in blackflies, namely, antimicrobial peptides (AMPs), proteases, phenoloxidases (POs) and haemolymph lectins. The AMPs included attacin-like molecules (23 kDa), lysozyme (14 kDa), cecropins and defensins (4–8 kDa). Antibodies raised against some of these peptides inhibited immune killing of *Onchocerca* sp. microfilariae in *Simulium* haemolymph [218]. The proteases were both serine and cysteine proteases, and it was postulated that some of these are infection-specific in response to both bacteria and microfilariae and may have been involved in PPO activation [173]. Levels of PO in microfilariae-infected blackflies were reduced compared with controls, possibly due to binding to the parasites and internal vector tissues. The haemolymph lectins recorded by Ham et al. [173] were hypothesised to be involved with PO/PPO in the recognition of non-self resulting in signal transduction to the nucleus and the induction of genes [173]. More recently, a defensin and a cecropin were identified and characterised from the salivary glands of *Simulium bannaense* [172]. The defensin, *Siba*Def, had high antimicrobial properties against Gram-positive bacteria while the cecropin, *Siba*Cec, possessed potent activity against Gram-negative bacteria. *Siba*Cec also had low cytotoxicity towards mammalian cells, neutralised LPS and exhibited strong anti-inflammatory activity [172]. Finally, progress has been made in identifying the composition of the bacteriome of blackflies. The phylum Proteobacteria predominates in the blackfly core bacteriome with *Wolbachia* being the most dominant genus [175]. Importantly, the infection status of the blackflies had a significant association with the abundance of certain bacterial genera such as *Serratia* [175]. With further research, these bacteria have potential for the development of innovative control techniques including paratransgenesis.

### 5.3. Sandflies (Table 1 and Table 3)

Approximately 500 species of sandflies (phlebotomine) have been described with more than 90 transmitting leishmaniasis. Species and subspecies of *Phlebotomus* in the Old World and *Lutzomyia* in the New World are the main vectors of human leishmaniasis [219]. There are numerous papers on various aspects of sandfly biology with some consideration of the role of the vector immune response as a determinant of infection by *Leishmania* parasites. Examples of relevant publications on sandfly innate immunity include Dillon et al. [176], Boulanger et al. [220], Telleria et al. [8], Coutinho-Abreu et al. [221], Kykalová et al. [222], Omondi et al. [177] and Campolina et al. [223]. Dillon et al. [176] undertook an analysis of expressed sequence tags (ESTs) derived from a whole-body cDNA library from *Lutzomyia longipalpis* sandflies, some of which were infected with *Leishmania infantum*, and revealed putative proteins involved in the barrier function of the vector gut, digestive physiology and the immune response. The immune factors identified included Gram-negative-binding proteins, galectins, thioester proteins, scavenger receptors, signaling pathway factors, serpins, caspases and peroxidases. The detection of these proteins indicated the presence of an active innate immune system in sandflies capable of interacting with invading parasites/pathogens like *Leishmania.* Evidence for this has also been provided by transcriptomic studies of changes in immune gene expression following infection with *Leishmania*. The genes affected included members of the Toll, Imd and JNK pathways and the antioxidants catalase, glutathione s-transferase, superoxide dismutase and peroxiredoxin, controlling ROS levels [8]. The upregulation of the Dorsal and Relish genes, that are positive modulators of the Toll and Imd pathways, respectively, also occurred following *Leishmania* challenge and led to increases in the expression of the AMPs attacin, cecropin and defensin 2 at different time points in the sandfly LL5 cell line [127]. In contrast, *Leishmania*-infected *L. longipalpis* showed no significant changes in ROS gut levels compared with controls. Since reductions in numbers of *Leishmania* in the sandfly gut occur following silencing of the sandfly antioxidant, catalase, this may indicate manipulation of vector antioxidative elements by the parasite [127].

The above implies the involvement of components of the sandfly innate immune response following *Leishmania* infections. Consideration, however, of the published work leads to some contradictions in observations recorded since minimal responses to the presence of *Leishmania* in the sandfly gut have also been published [221]. Similar inconsistences have also been noted in research on mosquito immunity in which the vector insects or parasites used were derived from different vector or parasite populations or used in alternative combinations. In addition, variations in insect physiological states or parasite developmental stages and the use of alternative sampling and analysis techniques can all affect the results [26]. *Leishmania*, in contrast to *Plasmodium* in mosquitoes, is confined to the gut of sandflies where it undergoes several developmental changes. The amastigotes in the blood meal develop into to weakly motile procyclic promastigotes in the peritrophic membrane, then to strongly motile long nectomonad promastigotes in the midgut lumen that transform into short nectomonad promastigotes, also called leptomonads, which eventually form the infective metacyclic stage which are regurgitated and transmitted during blood feeding [224]. All these various forms probably present unique molecular surface challenges to the sandfly immune system and result in variations in the vector response [8]. This emphasises the complexity of the sandfly–parasite association which is made even more complex by differences in the microbiomes of the vectors.

Studies of sandfly microbiomes have identified Gram-negative members belonging to the phylum Proteobacteria as dominant with the Gram-positive phyla Firmicutes and Actinobacteria also present. The Proteobacteria include *Serratia* and *Enterobacter* in the family Enterobacteriales and *Pseudomonas* as core taxa [8,26,225]. The role of the bacteriome in priming mosquito immunity and reducing *Plasmodium* infections has been described above (see Section 5.1 *Mosquitoes*), and similar reports exist for sandflies. For example, the effects of in vitro and in vivo co-cultivation for 24 h of each of 13 native bacteria isolated from *L. longipalpis*, at different developmental stages and physiological conditions, with promastigotes of *Leishmania infantum chagasi*, *Leishmania major*, *Leishmania amazonensis* and *Leishmania braziliensis* were analysed. After co-cultivation, a reduction in growth was recorded with all parasite species [223]. Also, with *L. longipalpis*, but infected with *L. infantum*, antibiotic-mediated perturbation of the midgut microbiome rendered sandflies unable to support parasite growth and metacyclogenesis. This suggests that an intact sandfly midgut microbiome is necessary for *Leishmania* development to its infective stage [226].

The reasons for any variations in experimental results has been discussed in detail in other papers [8,26,223] and confirm the complexity of the sandfly–*Leishmania* relationship. This complexity emphasises the need for further studies, for example, of the sandfly haemocytes (see Section 4.1 *Haemocyte Types*), of the parasite evasion processes [8,221,223,227], of the potential for development of paratransgenesis [26] and vaccines [23,228], and of the role of the vector microbiome co-egested with the parasites during sandfly feeding in the establishment of *Leishmania* in the mammalian host [23,227].

### 5.4. Tsetse Flies (Table 1 and Table 3)

Tsetse flies (*Glossina* spp.) are viviparous and include 30–33 species and subspecies and are usually divided into the Morsitans, Palpalis and Fusca groups which are particularly important medically and economically due to transmission of African trypanosomes in humans and animals [229]. An excellent review and original papers on tsetse fly innate immunity are provided by Matetovici et al. [12], while Weiss et al. [132] emphasise the importance of tsetse cellular immunity, as described above (see Section 4.2.2 Phagocytosis).

The *Glossina* genome was published in 2014 and is almost twice the size of the *Drosophila* genome containing ca. 12,308 protein-encoding genes [230]. The competence of the tsetse flies as vectors is determined by many factors such as nutrition, age, sex and symbionts [229,230]. *Glossina* has a reduced component of some humoral immunity-related genes, for example, some AMPs, lysozyme, C-type lectins, peptidoglycan recognition proteins (PGRPs), glucan-binding proteins, serine proteases and serpins, while other immune genes are expanded such as those encoding for the AMPs, attacin A and attacin B and for the thioester-containing protein (TEP) family [12,179,230]. In addition, tsetse EP protein, reactive intermediates of oxygen and nitrogen species (ROS, RNS), coagulation, melanisation, phagocytosis and the peritrophic membrane all contribute to the tsetse defence response [12,132]. The AMPs include the attacins A and B as well as cecropins A1, A2, B and C that are induced through the Toll and Imd pathways [12,179].

Particularly significant is the role of the symbiont *Wigglesworthia* in the development of the immune system in the tsetse larvae in order for the immune system to function normally in adult flies. In a basic simplification of the research by Weiss et al. [132,180], pregnant female tsetse flies were fed a diet containing tetracycline. The antibiotic removes all symbionts from the flies, resulting in aposymbiotic adult *Glossina morsitans morsitans* (*Gmm*^Apo^), with a severely compromised immune system without phagocytic haemocytes and with abnormal expression of immunity-related genes. Subsequently, these flies rapidly succumbed to infection with normally non-pathogenic *E. coli*. Furthermore, the process of immune system development can be restored in intrauterine *Gmm*^Apo^ larvae when their mothers receive a diet supplemented with *Wigglesworthia* cell extracts. Therefore, molecular components of *Wigglesworthia* have immunostimulatory activity within tsetse flies, and this represents a novel evolutionary adaptation that links an obligate symbiont with its host [132,180]. *Wigglesworthia* also enhances, in the gut of intrauterine tsetse larvae, odorant-binding protein 6 that stimulates the hematopoietic RUNX transcription factor, lozenge, causing larval haemocyte precursors to develop into functional crystal cells and initiate the melanisation cascade, via prophenoloxidase release [231], and haemolymph clotting. In *Wigglesworthia*-free tsetse flies cuticular wounds also fail to clot [180].

The above is a basic description of tsetse fly innate immunity since many other aspects of this process have been hardly mentioned. For example, the role of the PGRPs in tsetse immunity is important with the *Glossina* genome containing six PGRP genes, four in the long (pgrp-la, -lb, -lc, -ld) and two in the short (pgrp-sa, -sb) subfamilies [230]. Of these, RNAi silencing of PGRP-LC in *G. morsitans* suppresses the Imd pathway, resulting in a strong inhibition of attacin expression and an enhancement of midgut trypanosome infections [12,232]. Tsetse PGRP-LB, however, functions to degrade peptidoglycan from microbes, preventing an overactive immune response, and avoids damage to the essential Wigglesworthia symbionts in the tsetse bacteriome [12,232]. PGRP-LB, together with the other AMPs, has strong trypanocidal activity against procyclic and bloodstream trypanosomes [233]. In addition, the peritrophic matrix forms the first barrier to the parasites and determines the immunological detection of the invader. In summary, this immune detection and the Imd-pathway-associated PGRP-LB, the AMP attacin and the cellular immune system are key components of the tsetse fly/trypanosome interaction that leads either to parasite establishment or elimination from the midgut [12,132].

### 5.5. Lice (Table 1 and Table 3)

There are about 4000 species of lice but only 3 species infest humans, namely, the body louse, *Pediculus humanus* (=*P. humanus humanus*), the head louse, *Pediculus capitis*, and the pubic or crab louse, *Pthirus pubis* [56]. The body louse and head louse are closely related and may belong to the same species. The human body louse, *P. humanus*, vectors *Rickettsia prowazekii*, causing epidemic typhus, as well as *Borrelia recurrentis*, causing epidemic relapsing fever, and *Bartonella quintana*, the causative agent of trench fever [56]. Body lice may also be the vectors of plague caused by *Yersinia pestis* [234]. It is generally regarded that body lice act as vectors of these diseases and not the head lice although there is increasing evidence that head lice may be vectors too [234].

Lice are ectoparasites with infections often spread from the faeces or from crushed insect bodies, with blood meals taken several times per day by members of the suborder Anoplura [56]. The genome sequence of the body louse and a symbiont were published in 2010 [235] and showed that the body louse has the smallest genome of any hemimetabolous insect reported up to 2019 [181]. Comparisons have been made between the immune systems of body and head lice to identify any differences resulting in the primary vector role of the former and not of the latter [115,129]. For example, following bacterial challenge with *Bartonella quintana*, several genes in the body lice are downregulated compared with head lice and the bacteria multiply at a higher rate in the body lice too [182]. A transcriptional analysis of the basal immune response of the guts of body lice and head lice showed that the transcript levels of important immune genes, such as the peptidoglycan recognition protein and defensins, were reduced in body lice while the defensin 1 transcription following *B. quintana* oral infection was only upregulated in head lice [182]. Furthermore, the level of ROS produced by epithelial cells was significantly lower in body lice [182]. These results seem to indicate that the higher vector capacity of the body lice may be related to the reduced expression of certain key immune genes [129,181,182]. Immune genes associated with the Toll pathway have been identified in lice including those for the AMPs, scavenger receptor A, fibrinogen-like protein, and Spätzle. Lice also lack an Imd gene but retain some other Imd pathway genes [115] and can still respond to Gram-negative bacteria like *E. coli* [129]. Regarding symbiotic bacteria, both body and head lice have an endosymbiont, *Candidatus* Riesia *pediculicola*, in special enlarged midgut cells called mycetocytes forming the mycetoma and producing certain B vitamins not present in the blood meal [236].

### 5.6. Fleas (Table 1 and Table 3)

There are ca. 2574 species of fleas with 16 families and 238 genera, but only a minority are synanthropic, i.e., live intimately with humans [237]. Common synanthropic species are *Pulex irritans* (human flea), *Ctenocephalides felis* (cat flea) and *Xenopsylla cheopis* (rat flea). Diseases transmitted by fleas include plague, caused by *Yersinia pestis*, murine typhus (endemic typhus, *Rickettsia typhi*), rural epidemic typhus (*Rickettsia prowazekii*) in the USA, spotted fever agent *Rickettsia felis* and *Bartonella* spp., including *Bartonella henselae*, the agent of cat-scratch disease. *P. irritans* is an inefficient vector of plague bacteria, *Y. pestis*, compared with the rodent flea, *X. cheopis*. Furthermore, fleas also carry the helminths *Dipylidium caninum* and *Hymenolepis diminuta* that, respectively, are parasites of carnivores and rats. Finally, tungiasis in the tropics is a human disease linked to the parasitism of humans by fleas [238].

Often, pathogen transmission by fleas occurs orally through regurgitation of blood meals or from contaminated faeces [238]. The outcome of infection of fleas by bacterial pathogens also varies according to both the species of the flea and of the infecting bacteria [239]. Thus, *Y. pestis* is usually confined to the gut of different flea species while rickettsial pathogens, such as *R. felis*, may penetrate the midgut epithelium to migrate rapidly through the haemocoel to the salivary glands [240]. Therefore, any description of the immune response of fleas to bacterial invasion needs to consider events occurring in the midgut, haemolymph and salivary glands. This process may be complicated in *C. felis*, and probably *X. cheopis*, by the widespread gene duplication in the genome with genome sizes ranging from 433–551 Mb for individual fleas in different populations [241]. Therefore, every cat flea has a unique genome sequence with gene duplication as a source of genetic innovation creating problems in gene-targeting pest control measures and complicating comparative transcriptomics analysis [241].

In the flea midgut following an infected blood meal, the bacteria need to avoid being excreted and so they form biofilm aggregates or bind to the gut by receptor/ligand interaction [9]. For example, in *X. cheopis*, *Y. pestis* forms a biofilm which blocks the proventricular valve causing regurgitation of the flea gut contents and pathogen transmission into the wound [183,242]. Studies on the gut transcriptome of *X. cheopis* showed that the initial immune response to *Y. pestis* infection was the upregulation of AMPs. Coincidentally, Relish, an NF-κB-like transcription factor controlling gene expression in the Imd pathway, was also upregulated in infected fleas. In addition. the peptidoglycan recognition proteins (PGRP-LC and PGRP-LB), which are also activators and regulators of the Imd pathway, were upregulated too. The AMPs detected included two attacins and a coleoptericin-like peptide [183]. Gene expression of antibacterial ROS, however, was limited, so that that *Y. pestis* fails induction of a strong initial ROS response in *X. cheopis* [183]. However, previously, ROS have been described in response to *Y. pestis* infection in the gut of *X. cheopis* [130,183] and this illustrates either the variable nature of flea immunity and/or differences in the experimental protocols used. Cactus, the negative regulator of Toll, was upregulated by infected and sterile blood meals, so the Toll pathway probably does not participate in the flea immune response to *Y. pestis* [183]. Other transcripts modulated in the midgut by infection and/or a blood meal include serine proteases in *C. felis* midguts infected by *R. typhi* [184], while serpins and PPO activators have also been detected [9].

Regarding the invasion of the flea haemocoel, salivary glands and other tissues by rickettsial pathogens, such as *R. felis* and *R. typhi*, these organs have also been shown to have active immune responses to bacteria [111,240,243]. For example, utilising model bacteria injected into the haemolymph of the cat flea. *C. felis*, Muñoz et al. [111] found that the haemolymph had enhanced antibacterial activity and increased numbers of haemocytes so that additional studies with pathogens were justified. Likewise, studies on *C. felis* injected with *R. felis* and using PCR showed that defensin-2, glycine-rich AMPs and several flea antigens were modulated by rickettsial infection [240]. Finally, apart from the bacterial pathogens transmitted, fleas are also known to have other bacteria in their microbiomes, but information is limited except that a diverse range of *Wolbachia* strains have been reported with unknown interactions with flea immunity and vector competence [244].

### 5.7. Triatomines (Table 1 and Table 3)

There are ca. 150 species of triatomine bugs [245,246] distributed mainly in South and Central America and the southern USA. Most triatomine species are capable of vectoring the haemoflagellate protozoan parasite, *Trypanosoma cruzi.* In contrast, the metacyclic form of the closely related species *Trypanosoma rangeli* is reported to be mainly confined to *Rhodnius* species [247]. *T. cruzi* infection occurs after a blood meal from the human host by the triatomine vector which results in transmission of Chagas disease, an often fatal condition of the heart and gut [245]. The *T. cruzi* development is confined within the gut, from stomach to rectum, while *T. rangeli* invades the haemocoel and haemocytes before colonising the vector salivary glands [248]. For *T. cruzi*, *Rhodnius*, *Triatoma* and *Panstrongylus* species are epidemiologically most important in Latin America, as they colonise peridomestic and domestic areas close to humans and their animals [246,249].

There are considerable advantages of using triatomines for research into the immune reactions of insect vectors to their parasites and pathogens. First, the large size of many triatomines means that tissue samples, such as haemolymph, are easily obtained. For example, the most commonly researched triatomine, *R. prolixus*, grows up to 34 mm in length which is ca. 10 times the size of sandflies and fleas and at least 5–7 times that of lice, blackflies and mosquitoes (personal observations). Secondly, *R. prolixus* is relatively easily cultured, and there is already much information on its physiology gleaned from decades of research by entomologists of this vector as a model insect [250].

In this Special Issue, Schaub et al. present a detailed overview of “triatomine innate immunity against parasites” so only a basic description of triatomine humoral immunity is given here. Emphasis will therefore be given to some of the more recent research since the publication of the *R. prolixus* genome in 2015 [185]. In addition, many details of research progress in understanding triatome immune responses following infection are also given in a number of the most up to date reviews [21,24,27,135,137,138,186,246,251,252,253,254].

The humoral immune defences of triatomines have a number of factors/processes produced by the haemocytes, fat body, gut and other tissues, including AMPs, ROS, NO, PPO, melanisation, lysins, lectins and enzymes. All of these come together to combat would-be invaders, denying infection so that only a minority of vectors usually succumb to transmit disease.

Regarding the AMPs, an arsenal of these has been detected in triatomines including defensins A, B and C, lysozymes A and B, prolixicin, attacin, diptericin and trialysin [137]. The PRGPs recognise the PAMPs on the surface of the invaders and the Toll, Imd and JAK/STAT pathways are activated to induce the expression of the AMPs. Previously, there was some doubt about whether the Imd pathway in *Rhodnius* was fully functional, but using knockdown experiments of one of the PGRP genes reduced AMP expression induced by Gram− bacteria and confirmed the role of the Imd pathway in AMP expression [119]. There has also been interesting research on *Triatoma pallidipennis* in which the Toll and Imd pathways were silenced separately or together and then insects challenged with bacteria. This resulted in insect survival rates of ca. 62–73% when silencing occurred separately, but this fell to ca. 36% for *E. coli* and 41% for *Micrococcus luteus* after silencing both pathways. This indicated that the Imd and Toll pathways participate jointly to eliminate Gram+ and Gram− bacteria [139]. Challenging with T. *cruzi* or *T. rangeli* and monitoring parasite numbers might have yielded interesting results regarding the participation of the Imd and Toll pathways in immune reactions towards parasites.

Using quantitative proteomics for the analysis of the *R. prolixus* haemolymph from 6 to 24 h following *T. cruzi* infection also identified novel proteins with possible roles in immune reactivity to parasites [24]. These included an immunoglobulin I-set domain-containing protein (T1HCN4) for the first time in triatomines and of unknown function but which like haemolin might be involved in inhibition of haemocyte aggregation [24]. Another protein identified was a C1q-like protein which was strongly induced by *T. cruzi* infection compared with uninfected blood alone and may also be involved in the vector immune response to parasitisation [24].

A complex of interacting factors from the *R. prolixus* vector as well as from the invading trypanosomes determine the outcome of infection and the subsequent survival or killing of the parasites [135,136,137,138]. One key factor that seems to be involved in the invasion and survival of *T. cruzi* in *R. prolixus*, and other vector insects, is NO, with reactive nitrogen species (RNS) resulting from activation of nitric oxide synthase (NOS) to yield NO. It has been shown that NOS gene activity and NO production are specifically modulated in *R. prolixus* according to the nature of the invading organism so that differential responses occur following infection with *T. cruzi* and *T. rangeli* [255,256,257]. Results of Whitten et al. [256] together those of Baptista et al. [258] showed an augmentation of NOS gene expression by treatment with L-arginine, an activator of NO, and a reduction of NOS expression with L-NAME, an inhibitor of NOS. These modulations coincided not only with different levels of parasites in the gut but also with changes in levels of phenoloxidase, superoxide anion and antimicrobial activity but also with reductions in intestinal microbes. Such reductions in numbers of bacteria following infection with *T. cruzi* and *T. rangeli* were confirmed in a recent metagenomic shotgun sequencing study of the gut microbiome of *R. prolixus* [253]. These studies confirm several aspects of the complexity of the triatomine immune response with no real explanation of the factors governing these changes. It is essential that the responses occur early on in the invasion of the vector insect by the parasites since the anterior gut is a hostile environment with the majority of parasites often failing to infect the host insect. It has previously been postulated that surface components of *T. rangeli* directly or indirectly cause rapid modulation of the insect vector immune system after parasite invasion into the haemocoel [259]. In addition, *T. cruzi* has been reported to spontaneously secrete extracellular vesicles [260]. Such vesicles have been shown in humans to play key roles in pathological and physiological functions [261]. In protozoan parasites causing malaria and Chagas disease, there is also evidence that extracellular vesicles enhance growth and promote transmission, helping to avoid host immunity and to modulate the microenvironment [261].

Potential factors modulating the complex immune interactions between the vector insect and trypanosome parasites are the prostaglandins (PGs) and other eicosanoids. These molecules have been shown to play important roles in insect innate immunity [142,186,187,262,263] but, with notable exceptions, seem to have had less attention recently. This may be due to the fact that cyclooxygenase (COX) genes responsible for converting C20 polyunsaturated fatty acids (PUFAs) into PGs seem to be absent from published insect genomes [263]. There is now confirmed evidence that eicosanoids mediate both cellular and humoral immunity in insects including much research in *R. prolixus* and some in mosquitoes [187,264]. Significantly, eicosanoids have their effects early in the immune response and are involved in phagocytosis, nodule formation, clotting, haemocyte chemotaxis and aggregation, ROS formation, melanisation by PPO, mediation of AMP gene expression in both Toll and Imd pathways, interactions with the fat body and Toll/Imd signaling to trigger NO to activate PLA_2_ (phospholipase A_2_) and synthesise eicosanoids [142,264]. The research by Barletta et al. [187] with *An. gambiae* mosquitoes invaded by *Plasmodium* parasites is classic and beautifully designed and executed and confirms the role of the midgut production of prostaglandin E2 to attract haemocytes and induce long-term systemic cellular immune responses to infection by *Plasmodium* parasites (see also Section 6.3.6 *Immune Priming* for more details). Finally, it is well known in vertebrates that eicosanoids effect the expression of inducible NO (iNOS) and NO production [265] so much can also be gained by reading this literature.

## 6. Recognition, Signalling and Priming in Vector Innate Immunity 

Previously, mention was made of recognition, signaling, RNA inhibition and priming of vector immune responses to invading pathogens/parasites but little detail was provided of these processes. In this section, additional consideration is given to these responses with reference to *Drosophila* which provides a comparative model and guide for investigations into these processes in vector insects by assumptions that the innate immune responses of *Drosophila* are well conserved. These approaches include identification of orthologous genes, quantifying transcript abundance of these genes after pathogen challenge and RNA interference of key pathway regulators combined with quantifying the effect of this gene silencing on pathogen abundance in the manipulated vector. While for some vector insects the data underlying these assumptions are consistent with the canonical pathways in *Drosophila*, for others, such as triatomines, the available evidence points to the existence of non-canonical signaling pathways, as discussed below. 

### 6.1. Pathogen Recognition

As in other animals and plants, insects solve the conundrum of recognition of self versus non-self with the help of PRRs. These recognise PAMPs that are not features of insect cells, thus enabling discrimination between self versus non-self. These PAMPs are typically components of the cell surface of parasites/pathogens and can be categorised as polysaccharide PAMPs such as peptidoglycan (PGN), lipopolysaccharide (LPS), capsular polysaccharide, β-1,3-glucan and zymosan. Apart from LPS, other lipid-containing PAMPs include lipoteichoic acid and lipoarabinomannan, while proteinaceous PAMPs include capsid proteins and flagellins. Finally, pathogen-released nucleic acids are additional PAMPs, composed of viral RNA, both double- and single-stranded, and DNA [266].

PAMPs alert the insect to a possible infection, triggering the innate immune response. PRRs are the sensing proteins that initiate this response with some PRRs functioning to directly elicit responses such as phagocytosis and the PPO cascade, whereas others trigger signal transduction pathways that regulate immunity. We describe the principal PRR protein families involved in vector insect immunity; other candidate PRRs are reviewed elsewhere [267,268].

### 6.2. Pattern Recognition Receptors (PRRs)

#### 6.2.1. Peptidoglycan Recognition Proteins (PGRPs)

PGRP is a fundamental PRR conserved from insects to mammals, regulating the immune response to bacteria. Insects can express multiple PGRPs with each regulating specific immune responses. For example, in *D. melanogaster*, two extracellular PGRPs, PGRP-SA and PGRP-SD, recognise bacterial Lys-type PGN and stimulate the Toll pathway after infection by Gram-positive bacteria, with the latter also upregulating the Imd pathway [269,270,271,272]. The Imd pathway is also stimulated by extracellular PGRP-LE and a membrane-bound PGRP-LC [273,274]. Aside from eliciting these responses, PGRP-LE also participates in the PPO cascade [275,276] converting prophenoloxidase (PPO) to active phenoloxidase (PO) that leads to localised wound healing and melanisation. To counter cell-invasive pathogens such as *Listeria monocytogenes*, intracellular PGRP-LE also stimulates autophagy [277].

In an in silico study conducted using the genome of *An. gambiae*, approximately 150 putative PRRs were identified, the majority of which were found to be secreted proteins having adhesive domains to interact with PAMPs. These PRRs were found to cluster as members of large families of genes [278].

#### 6.2.2. Immunolectins 

Typical insect immunolectins (IMLs) are calcium-dependent C-type lectins that contain either single or dual carbohydrate receptor domains. They can bind to LPS, lipoteichoic acid and β-1,3-glucan and participate in a variety of immune responses, including encapsulation, phagocytosis, nodulation and activation of the PPO cascade [279] Another important family of carbohydrate-binding proteins in the mosquito’s innate immune response are the fibrinogen-related proteins (FREPs = FBNs). In *An. gambiae*, they are the largest pattern recognition protein family consisting of 59 putative members, most of which showed immune responsive transcription when challenged by infection with bacteria, fungi or *Plasmodium* [280,281]. RNAi-mediated gene-silencing assays showed that members of the FREP family play a central role in the innate immune response and maintenance of immune homeostasis in mosquitoes [282]. Thus, FBN9, FBN30 and FBN39 inhibit *Plasmodium* infection in *Anopheles* midgut epithelial cells [202]. In contrast, FREP1 functions as an anchor in the peritrophic membrane helping invasion of the mosquito midgut by *P. falciparum* ookinetes, with CRISPR-Cas9 knockout of *FREP1* mosquitoes resulting in suppression of infection by both oocysts and sporozoites [202].

#### 6.2.3. Thioester-Containing Proteins (TEPs) 

TEPs are secreted glycoproteins containing a highly reactive thioester motif that covalently binds to microbial surfaces [283] and function as opsonins, promoting phagocytosis, lysis and the PPO cascade [284]. Studies in *Drosophila* indicate that several different TEPs acting as PRRs can activate the immune Toll, Imd and JAK/STAT signaling pathways [285,286]. The mosquito, *An. gambiae,* encodes up to 15 TEPs, and of these, TEP1 plays important roles in (i) the phagocytosis of bacteria [287], (ii) the lysis of *Plasmodium* ookinetes [288] and (iii) the lysis and melanisation of entomopathogenic fungi [289]. TEP1 of *Ae. aegypti* limits infection by dengue virus and is required for activation of the Imd signalling pathway and AMP synthesis [290].

### 6.3. Signal Transduction Pathways

Pathogen recognition activates signal transduction pathways to amplify and coordinate the immune responses including synthesis of antimicrobial factors. PAMP recognition by PRRs leads to activation of protein kinases, proteases and other enzymes in pathways that ultimately act on core transcription factors that, once translocated into the nucleus, activate expression of immune-related genes. Different immune signaling pathways have been described in insects, including Toll, Imd, JAK/STAT, JNK and RNAi [267,291]. The JAK/STAT pathway regulates immune response genes related to viral and bacterial infections and the RNAi pathway mainly controls virus replication. The Toll and Imd pathways are inflammatory responses that activate expression of a wide spectrum of AMPs through the activation of the nuclear factor-kappa B-like (NF-κB-like) transcription factors. The activated NF-κB is translocated from the cytosol into the nucleus of the cell, binding to specific sequences of DNA and inducing the transcription of AMP genes [292].

#### 6.3.1. The Toll Signalling Pathway

Insect Toll receptors perform critical functions in both embryogenesis and innate immunity. Toll receptors have an extracellular leucine-rich repeat region, a transmembrane domain and an intracellular Toll/interleukin-1 receptor (TIR) domain and are activated by a complementary family of six cytokine-like molecules called Spätzle (Spz) [293]. The terminal proteinase Spätzle-processing enzyme (SPE) has trypsin-like specificity and cleaves the Spz precursor. The released C-terminal polypeptide can dimerise and bind to Toll receptors in the membrane [294], triggering a conformational change to the cytoplasmic TIR domain so that it can recruit a heterotrimeric complex of three proteins: MyD88, Tube and the kinase Pelle [295]. The protein kinase Pelle promotes the phosphorylation and degradation of Cactus, a cytoplasmic ankyrin-repeat-containing protein that retains NF-κB family transcription factor Dorsal and Dorsal-related immune factor (Dif) in the cytoplasm [295,296]. After degradation of Cactus and translocation to the nucleus, Dorsal and/or Dif can then activate expression of antimicrobial factors such as AMPs. In *Drosophila*, the Toll pathway regulates both cellular and humoral immune responses and these may respond to an interplay between the Toll pathway and eicosanoid signalling (see below).

In *An. gambiae*, the Toll pathway controls expression of the antiparasitic proteins TEP1, a thioester-containing protein, and leucine-rich repeat immune protein 1 (LRIM1). Basal-level expression of these factors and their secretion from haemocytes, resulting from an immune-priming mechanism, reduces initial parasite infection [297]. Moreover, post-infection, the expression of these factors increases, presumably to modulate parasite numbers. Experimental RNAi of Cactus, the inhibitor of Relish1 (the NF-κB gene controlling the Toll response in *An. gambiae*), results in overexpression of these factors, completely blocking parasite development in the mosquito. Commensal *Asaia* bacteria can prime *Anopheles* immunity to limit subsequent *Plasmodium* infection by upregulating expression of TEP1 [213].

#### 6.3.2. The Imd Signalling Pathway 

In insects such as flies, mosquitoes and beetles, PGRPs including PGRP-LC and PGRP-LE form complexes with DAP-type PGN of Gram-negative bacteria. Recognition of the PGRPs results in the recruitment of the Imd protein [298] and then an intracellular signaling cascade is activated by the interaction of Imd with other intracellular signaling proteins [299]. This leads to the translocation of the transcription factor Relish into the nucleus where it binds to the transcription response elements of AMP genes and promotes their expression [300].

In *Aedes* mosquitoes, the Imd pathway is activated by bacteria and *Plasmodium*, and an indirect effect of the Imd pathway has been shown on viral load [301]. Although the Toll and JAK-STAT pathways contribute to the immune response against *Plasmodium*, the Imd pathway is considered the primary response [302]. The NF-κB transcription factor in the Imd pathway is represented by the protein Rel-2. It plays the central role in Imd signalling and regulates a major AMP, cecropin1. Multiple studies have shown that Caspar functions as a negative regulator of the Imd pathway and that the immune response against *Plasmodium* could be exaggerated by the silencing of the Caspar gene [302].

In sandflies, gene silencing via RNAi of Caspar [303] led to a reduction in the *Leishmania* population in the gut of *L. longipalpis*, while the knockdown of Relish (the transcription factor gene of the Imd pathway) resulted in the increase in *Leishmania* and bacteria in *P. papatasi* [304].

The cat flea *Ctenocephalides felis* contains homologues to *Drosophila* Imd pathway genes and inactivation of Relish or Imd genes increases *Rickettsia typhi* burden in the gut of infected fleas [305]. However, mutant strains of *Yersinia pestis* that are highly sensitive to AMPs in vitro exhibited normal growth in the digestive tract of fleas, which suggests that the presence of this pathogen in vivo does not automatically induce AMP gene expression [306], indicating that fleas are permissive hosts of this bacterium.

Tsetse flies have a full complement of Imd signalling pathway genes and this pathway responds to trypanosome infection. RNAi silencing of the Imd pathway transcriptional factor Relish (GmmRel) prevents the activation of the pathway, blocking the expression of both attacin and cecropin AMPs and increasing the establishment of trypanosome infection in the tsetse midgut [232,307].

Some insect species appear to lack key Imd signalling components. For example, the triatomine *Rhodnius prolixus* lacks both Imd and Kenny genes [136], and the function of other identified candidate Imd pathway genes has yet to be determined. RNA interference, inhibition studies and qRT-PCR experiments implicate remaining Imd pathway genes, such as Relish, being involved in a possible non-canonical Imd-Toll pathway regulating expression of AMPs as a result of challenges by either Gram+ or Gram− bacteria [308,309,310]. Infection with the parasite *T. cruzi* can result in increased AMP expression that, in turn, impacts competing bacterial numbers in the midgut, enabling the parasite to establish an infection. 

Lice also lack an Imd gene but retain some other Imd pathway genes [115]. They also have a remarkably small complement of effector AMP genes, namely two defensin genes. Challenge with Gram-positive *Staphylococcus aureus* resulted in increased expression of these two AMP genes in the absence of upregulation of Toll or Imd pathway genes [311]. However, no responses were detected after challenges with Gram-negative *E. coli*. These results suggest that lice are somewhat permissive to bacterial infection, underlining their role as important vectors of bacterial pathogens. This functional crosstalk across Imd and Toll pathways may be more common than generally recognised among different insect species and could be an evolutionarily intermediate step towards the loss of Imd-related genes [312]. This hypothesis suggests repeated and independent losses of Imd pathway components occurring during arthropod evolution. Alternatively, hemimetabolous insects and other arthropods that characteristically do not liberate their gut bacteria during metamorphosis may possess an ancestral incomplete (relative to holometabolous vectors and *Drosophila*) Imd pathway. For example, the tick *Ixodes scapularis* lacks genes encoding upstream regulators of the Imd pathway, including Imd, and FADD [313]. Despite the absence of these regulators, the remaining Imd pathway is active against infection by tick-borne pathogenic bacteria such as *Borrelia burgdorferi* and is triggered by cellular stress responses centring on the evolutionary conserved unfolded protein response (UPR) [314]. We speculate that this could also be true for hemimetabolous vector insects.

#### 6.3.3. The JAK/STAT Pathway 

The JAK/STAT signaling cascade takes place in the fat body and gut cells and is elicited upon injury and microbial infections. It is activated in response to unpaired (Upd) cytokines released by haemocytes [315,316]. In *Drosophila*, Upd3 binds to the membrane Domeless receptor [316] which dimerises, causing phosphorylation of the Janus kinase specific to *Drosophila* called Hopscotch [316,317,318]. In turn, this allows phosphorylation at conserved tyrosine residues of the signal transducer and activator of transcription (STAT) transcription factor. Phosphorylated STAT dimerises and translocates to the nucleus where it activates expression of target genes [316,318]. These genes include TotA, Tep1 and Tep2 [318] whose products are released into the haemolymph and promote wound healing, resistance to stress and phagocytosis [287,299]. The JAK/STAT pathway contributes to cellular immune responses, such as haemocyte differentiation, and renewal of intestinal epithelial cells. Aberrant activation of the JAK/STAT pathway can result in nodulation in which the PPO cascade is activated [319]. STAT knockdown by RNAi increased the number of *Plasmodium vivax* in the midgut of *Anopheles aquasalis* and enhanced *P. berghei* and *P. falciparum* infection in the digestive tract of *An. gambiae* [320,321].

The JAK–STAT pathway in mosquitoes also responds to viral infections. Knockdown of the Domeless and Hop genes in *An. aegypti* enhances the burden of dengue virus (DENV), while silencing PIAS (a negative regulator of the JAK–STAT pathway) results in enhanced resistance to DENV infection [322]. STAT gene silencing mediated by RNAi reduced the expression of iNOS and favoured *Leishmania* growth in sandflies [323]. Based on gene conservation, other vectors have JAK/STAT pathways but their function in relation to pathogens has yet to be determined in detail. 

#### 6.3.4. JNK Signaling 

Jun N-terminal kinase (JNK) represents a subgroup of mitogen-activated protein kinases which are evolutionarily conserved in eukaryotic cells and activated by environmental stresses and inflammatory cytokines [324]. Upon activation, JNK phosphorylates the transcription factors Jun and Fos, leading to formation of the Jun/Fos dimer, which activates transcription of target genes. The JNK pathway is required for AMP gene expression in *Drosophila* [325]. JNK signalling is a key regulator in the mosquito *An. gambiae*, limiting *Plasmodium* infection [326]. DENV2, ZIKV or CHIKV activates the JNK pathway which reduces viral infection by inducing complement and apoptosis in the salivary glands of *Ae. aegypti* mosquitoes [327].

#### 6.3.5. Eicosanoid Signaling 

Eicosanoids are oxygenated metabolites of polyunsaturated fatty acids known to act as signaling molecules in animals. They are derived from arachidonic acid (AA) released from phospholipids by hydrolytic cleavage mediated by phospholipase A_2_ (PLA_2_). In *Drosophila*, the Imd signaling pathway can be elicited by exposure to bacterial LPS, activating expression of AMPs, and this response can be suppressed by exposure to specific PLA_2_ inhibitors [328]. As this suppression can be reversed by supplementation with AA, this suggests that eicosanoids are important mediators for relaying the Imd response. Eicosanoids are implicated in both humoral and cellular immune responses, including nodulation, the PPO cascade and haemocyte spreading [186].

In *An. gambiae*, a receptor for the eicosanoid prostoglandin E2 (PGE_2_) is expressed in oenocytes, differentiated haemocytes implicated in the production of phenoloxidases leading to melanisation responses. PGE_2_ regulates both the PPO cascade and also AMP expression in these cells to limit both bacterial infection and *Plasmodium* oocyte survival [329]. In *Ae. aegypti*, experimentally reduced prostaglandin synthesis is associated with decreased expression of components of the Toll and Imd immune pathways, thereby rendering mosquitoes more susceptible to both bacterial and viral (dengue) infections [330]. It is suggested that prostaglandins control the amplitude of the immune response to guarantee an efficient pathogen clearance. In *R. prolixus*, the survival of the parasite *T. rangeli* in the haemolymph involves the modulation of eicosanoid production (the hormone ecdysone also plays a crucial role in parasite survival in the haemolymph). The parasite inhibits phagocytosis by modulation of the phospholipase A2 and platelet-activating factor (PAF)–acetylhydrolase activities [331]. The enzymes have key roles in eicosanoids and PAF pathways, respectively. Exogenous supplementation of AA prevented parasite infection.

#### 6.3.6. Antiviral Immunity

Viral infections can induce Toll, Imd and JAK/STAT pathways in different insect species, although details on how specific viruses are sensed in this context is unclear even for *Drosophila*. Infection with *Drosophila* C virus (DCV) leads to induction of 130 genes, with many of these dependent on the *hop* gene encoding the JAK kinase [332]. Consequently, *hop* mutant flies are very susceptible to DCV infection. In addition, DCV can induce the Pelle-dependent nuclear translocation of the Toll-pathway-specific NF-κB transcription factor, Dorsal [332]. A functioning Toll pathway is required for resistance to *Drosophila* X virus [332], and the Imd pathway is implicated in resistance to infections by Sindbis virus [333]. In *Ae. aegypti*, both Toll and Imd pathways are stimulated by the endosymbiont *Wolbachia*, and this immune priming is crucial in the subsequent inhibition of dengue infection (DENV) [334]. In addition, the JAK/STAT pathway regulates innate immunity during DENV infection of *Ae. aegypti* by upregulating the expression of JAK, STAT and DENV response factors 1 (DENV-1) and 2 (DENV-2) [335]. Suppression of Zika virus (ZIKV) infection in *Ae. aegypti* requires the activation of both the Toll and JAK/STAT pathways [336]. RNA interference is also a major immune pathway for limiting arbovirus infection in mosquitoes. Disruption of this defence by CRISPR/Cas9-based knockout of the Argonaute (Ago) gene, encoding the major component of the RNA-induced silencing complex (RISC), impaired the mosquitoes’ ability to degrade arbovirus RNA, leading to hyper-infection accompanied by cell lysis and tissue damage [337]. The recently characterised cGAS-STING pathway in *Drosophila* [338] is required to control viral infections, for example, by DCV and cricket paralysis virus (CrPV), but we are not aware of studies on this pathway in vector insects.

#### 6.3.7. Immune Priming

As described herein and by numerous previous papers, exposure of insects for the first time to invading organisms, like parasites or pathogens, activates an effective immune response, but now evidence indicates that upon second exposure to the same organism, a more rapid and elevated immune response occurs. This process is called immune priming and has the hallmarks of acquired immunity in vertebrates but without the presence of specific memory cells and lymphoid tissue [65,339,340,341,342,343,344]. The possibility of immune priming in insects is not new and was shown by Karp and his co-workers, many years ago, using grafting and bacterial immunisation experiments [339,340]. The priming effect can be short- or long-term and can be pathogen-specific or non-specific [344]. Priming can also be prolonged throughout different developmental stages so that primed larvae can enhance adult immunity [345]. In addition, transgenerational immune priming also occurs by which the adults transfer immune effectors like AMPs or microbial components to the eggs and produces immune priming in the next generation. This subject is dealt with in detail by an excellent article by Andreas Vilcinskas [342].

Except for mosquitoes, however, much of the work on insect immune priming has been carried out with non-vector insects. In *Drosophila*, for example, a recent study with *Enterococcus faecalis* for immune priming showed that a low dose primes flies to survive longer with a subsequent high-dose infection for at least 7 days. This increased survival did not result from a more efficient bacterial clearance [346]. In addition, phagocytosis was required for full priming, since decreased priming occurred after bead-blocking experiments and in flies deficient in *Eater.* Since primed flies, however, failed to clear bacteria more effectively than non-primed controls, the haemocytes must be playing a non-canonical role in the priming process. A clue to this was indicated by phagocytic reprogramming and transcriptional changes in the metabolic pathways of the phagocytes from primed flies [346]. Fortunately, the overview of Gomes et al. [344] on immune priming in *Anopheles* by *Plasmodium* provides more information about the role of phagocytes in this process. *Plasmodium* infection produces primed immunity to subsequent infections and is dependent on increases in circulating granulocytes that mediate immune memory [344]. This enhancement is permanent, with the gut microbiome needed for establishment and the memory of the priming process, and not resulting from the continuation of the initial response to infection. This priming process involves different tissues, and eicosanoids are shown to be involved as systemic signaling molecules (see Section 5.7 *Triatomines*, above). More details are provided in Gomes et al. [344]. Briefly, *Plasmodium* infection results in production of two haeme peroxidases which mediate the midgut synthesis of prostaglandin E2 (PGE2). The systemic PGE2 release, results in the production of a haemocyte differentiation factor (HDF), enhancing haemocyte development into granulocytes. Subsequently, ookinete invasion and damage of midgut epithelial cells induce a nitration process and the cells undergo apoptosis. The systemic release of the PGE2 attracts granulocytes to the basal midgut and when the haemocytes contact the nitrated surfaces, they produce microvesicles that result in mosquito complement to destroy the ookinetes [344].

## 7. Final Comments

There has been an explosion of the literature on insect immunity although, apart from mosquitoes and triatomines, the majority of vector insect species have been neglected. Herein, we give key examples and also the most relevant recent references and reviews on vector immunity. We have also cited papers on *Drosophila* for comparative purposes and some of the more original work on insect immunity to acknowledge the contributions of pioneers in this important field without whom insect immunity would still be regarded as an irrelevant branch of immunity. We apologise sincerely to authors whose papers have not been included. There is much work still required on vector insect interactions with parasites and pathogens, and areas needing particular attention are indicated. Recently developed innovative molecular techniques should facilitate progress in the study of neglected vector systems such as those of fleas, lice, sandflies and blackflies. After all, if scientists can now analyse the molecular biology and functioning of single haemocytes then insect vectors with limited numbers of blood cells will be open to investigation too. The results of such research should identify important molecules and processes which potentially could be the subject of new control measures.

## Figures and Tables

**Table 1:** The main insect vectors, their distribution and the diseases transmitted.

Vectors	Diseases	Pathogens	Distribution	At Risk
*Aedes*, *Anopheles*, *Culex*, *Mansonia*	Lymphatic filariasis	Nematode worms *Brugia* spp., *Wuchereria bancrofti*, *Dirofilaria* spp.	Tropical and subtropical regions of SE Asia, Central and South America, Africa, West Pacific	882 million
*Aedes*	Dengue	Flavivirus	Tropical, subtropical and spreading to Europe	3.9 billion in 129 countries
*Aedes*	Yellow fever	Flavivirus	Endemic in tropical regions of Africa and Latin America	900 million
*Aedes*	Chikungunya	Alphavirus	Tropical, subtropical and temperate regions	¾ of the world population at risk
*Aedes*	Zika	Flavivirus	The Americas, Europe, India and 89 countries	Over 2 billion at risk
*Anopheles* complex with 484 recognised species but *An. gambiae* carries the deadliest disease forms	Malaria	Protozoan parasite with 5 *Plasmodium* species	In 2021, the African region carried 95% of cases	Nearly half the world was at risk of malaria in 2021
*Culex* spp.	Arboviruses	West Nile virus, (both flaviviruses), Japanese encephalitis	USA, Canada, Caribbean, Central and South America, South East Asia and West Pacific	These and other arboviruses (Zika, etc.) risk emerging pandemics
Blackflies *Simulium* spp.	Onchocerciasis, river blindness	Vector-borne nematode worms, e.g., *Onchocercas volvulus*	99% in Africa but also foci in Brazil, Venezuela and Yemen	123 million
Sandflies *Phlebotomus* spp. and *Lutzomyia* spp.	Leishmaniasis	Protozoan parasite with more than 20 species	Africa, Americas (Brazil), Middle East, South Asia, Mediterranean	99 countries
Tsetse flies *Glossina* spp.	African trypanosomiasis	Protozoan parasites, *Trypanosoma brucei*	Sub-Saharan Africa	55 million people but controlled now and less than 1000 cases in 2022
Lice *Pediculus humanus* and *Pthirus pubis*	Louse-borne typhus and severe allergic reactions	Rickettsiae *Rickettsia prowazekii*	Epidemics in wars, prisons and refugee camps in colder regions	Global diseases of poverty
Fleas *Xenopsylla cheopis*	Plague	Gram-negative bacterium *Yersinia pestis*	Associated with close living in deprived areas with rat infestations	Global distribution
Triatomine bugs *Rhodnius* and *Triatoma* spp.	Chagas disease	Protozoan *Trypanosoma cruzi*	Mainly South, Central America but also North America now	70 million in the Americas

Modified from WHO [38] and Shaw and Catteruccia [39].

**Table 3:** Basic summary of some important aspects of the humoral immune factors identified in vector insects *.

Vectors	Signal Pathways	Antimicrobial Peptides	Melani -sation	Lectins	ROS/RNS	Symbionts	Additional Information
*Aedes*	Toll, Imd and JAK-STAT for dengue ^3^ and Toll for antifungal defence	Defensins, cecropins, dipterincins attacins and gambicins	A complex process involving haemocytes, PPOs, CLIP domain serine proteases, serpins, TEP1 thioester-containing protein, C-type lectins. Parasites have avoidance mechanisms ^6^	C-type lectins CTL4 and CTLMA2 are also involved in mosquito defences against *Plasmodium*, fungi and bacteria. They function independently to the Imd and TEP1 melanisation process. Present in haemolymph and saliva ^7^	ROS/RNS both involved in pathogen killing. ROS may be derived from toxic quinones during melanotic encapsulation and from mitochondria. NO production in midgut cells causes nitration and release of activator of TEP1 ^8^	Form part of a tripartite association of mosquitoes with vector immunity. Most attention has been on bacteriomes for use in paratransgenesis. Microbiomes can both defend against pathogens as well as enhance infections. More attention is needed on the fungal and viral components of microbiomes	417 ^1^ immune genes
*Anopheles*	Toll, Imd, and JAK-STAT. Imd for anti-*P. falciparum* defence ^4^	Defensins, cecropins, attacins and gambicins					380 ^1^ immune genes, FREP1 ^2^ aids parasite entry
*Culex*	Toll, Imd and JAK-STAT. JAK-STAT for anti-WNV ^5^	Defensins, cecropins and gambicins					500 ^1^ immune genes
Blackflies ^9^	?	Attacin-like, lysozyme, cecropins, defensins	PO/PPO and serine proteases present	Haemolymph lectins present	?	Limited studies of microbiomes. Proteobacteria dominant. *Wolbachia* present in parasites	Neglected group, but can be mass reared
Sandflies ^10^	Toll, Imd, JAK-STAT, TGF-β	Defensins, cecropins, attacins, lysozyme	? but sandfly eggs melanise	C-type lectins and TEPs	ROS/NO expression downregulated by Leishmania?	Proteobacteria dominant but role inconsistent	Large genome
Tsetse flies ^11^	Toll, Imd, JAK-STAT	Attacins, cecropins, defensin, dipterincin PGRP-LB	PO/PPO, serine proteases, and serpins present with clotting and melanisation	C-type lectins and enhanced TEP expression	ROS/NO expression in proventriculus and salivary glands	Wigglesworthia necessary for immune competence. Progress made using *Sodalis* in paratransgenesis	12,308 protein-encoding genes. Cellular immunity important
Lice ^12^	Toll and some Imd pathway genes present	Defensins 1 and 2	PPO gene present	?	ROS expression	Body and head lice have a *Candidatus* sp. endosymbiont producing vitamin B	93 immune genes with expression differences in body and head lice
Fleas ^13^	Imd but Toll needs to be confirmed	Defensin 2, two attacins, coleoptericin-like peptide	PPO activators present	Lectins present	ROS activity	Diverse *Wolbachia* symbionts present	Widespread gene duplication. Biofilm formation
Triatomines ^14^	Toll, Imd, JAK-STAT, eicosanoids	Defensins A, B and C, lysozymes A and B, prolixicin, attacin, diptericin and trialysin	PPO genes and melanisation	Haemolymph and gut lectins present	RNS and ROS present and manipulated by parasites	Many symbionts present and manipulated by parasites. Paratransgenesis candidates tested	13,840 protein-coding and 1505 non-protein-coding genes

* See text for details. ^1.^ [161]. ^2.^ [162]. ^3.^ [163]. ^4.^ [164]. ^5.^ [159]. ^6.^ [144,149,152,165,166,167]. ^7.^ [167,168]. ^8.^ [6,169,170,171]. ^9.^ [172,173,174,175]. ^10.^ [8,176,177,178]. ^11.^ [12,26,132,179,180]. ^12.^ [129,181,182]. ^13.^ [9,111,183,184]. ^14.^ [24,138,139,185,186,187]. ? indicates something unknown.

## Data Availability

The authors confirm that the data supporting the conclusions of this study are cited within the article.
